# Vasculogenesis in kidney organoids upon transplantation

**DOI:** 10.1038/s41536-022-00237-4

**Published:** 2022-08-19

**Authors:** Marije Koning, Sébastien J. Dumas, M. Cristina Avramut, Roman I. Koning, Elda Meta, Ellen Lievers, Loes E. Wiersma, Mila Borri, Xue Liang, Lin Xie, Ping Liu, Fang Chen, Lin Lin, Yonglun Luo, Jaap Mulder, H. Siebe Spijker, Thierry Jaffredo, Bernard M. van den Berg, Peter Carmeliet, Cathelijne W. van den Berg, Ton J. Rabelink

**Affiliations:** 1 Department of Internal Medicine - Nephrology, Leiden University Medical Center, Leiden, the Netherlands; 2 Einthoven Laboratory of Vascular and Regenerative Medicine, Leiden University Medical Center, Leiden, the Netherlands; 3 Laboratory of Angiogenesis and Vascular Metabolism, Department of Oncology and Leuven Cancer Institute (LKI), KU Leuven, VIB Center for Cancer Biology, VIB, Leuven, 3000 Belgium; 4 Department of Cell and Chemical Biology, Leiden University Medical Center, Leiden, the Netherlands; 5 Lars Bolund Institute of Regenerative Medicine, Qingdao-Europe Advanced Institute for Life Sciences, BGI-Qingdao, BGI-Shenzhen, Qingdao, China; 6 MGI, BGI-Shenzhen, Shenzhen, China; 7 Department of Biomedicine, Aarhus University, Aarhus, Denmark; 8 Steno Diabetes Center Aarhus, Aarhus University Hospital, Aarhus, Denmark; 9 Department of Pediatrics, Leiden University Medical Center, Leiden, the Netherlands; 10 Division of Nephrology, Department of Pediatrics, Erasmus Medical Center, Rotterdam, the Netherlands; 11 Sorbonne Université, IBPS, CNRS UMR7622, Inserm U 1156, Developmental Biology Laboratory, Paris, France; 12 Laboratory of Angiogenesis and Vascular Heterogeneity, Department of Biomedicine, Aarhus University, Aarhus, 8000 Denmark; 13 State Key Laboratory of Ophthalmology, Zhongshan Ophthalmic Center, Sun Yat-Sen University, Guangzhou, Guangdong People’s Republic of China; 14 The Novo Nordisk Foundation Center for Stem Cell Medicine (reNEW), Leiden University Medical Center, Leiden, the Netherlands

**Keywords:** Induced pluripotent stem cells, Stem-cell differentiation, Regeneration, Podocytes

## Abstract

Human induced pluripotent stem cell-derived kidney organoids have potential for disease modeling and to be developed into clinically transplantable auxiliary tissue. However, they lack a functional vasculature, and the sparse endogenous endothelial cells (ECs) are lost upon prolonged culture in vitro, limiting maturation and applicability. Here, we use intracoelomic transplantation in chicken embryos followed by single-cell RNA sequencing and advanced imaging platforms to induce and study vasculogenesis in kidney organoids. We show expansion of human organoid-derived ECs that reorganize into perfused capillaries and form a chimeric vascular network with host-derived blood vessels. Ligand-receptor analysis infers extensive potential interactions of human ECs with perivascular cells upon transplantation, enabling vessel wall stabilization. Perfused glomeruli display maturation and morphogenesis to capillary loop stage. Our findings demonstrate the beneficial effect of vascularization on not only epithelial cell types, but also the mesenchymal compartment, inducing the expansion of ´on target´ perivascular stromal cells, which in turn are required for further maturation and stabilization of the neo-vasculature. The here described vasculogenic capacity of kidney organoids will have to be deployed to achieve meaningful glomerular maturation and kidney morphogenesis in vitro.

## Introduction

Since the publication of protocols for the generation of three-dimensional (3D) kidney organoids from human induced pluripotent stem cells (hiPSCs)^1–4^, expectations regarding their potential for the modeling of kidney development and disease have been very high. Over the past few years, it has become increasingly clear that the level of maturation in vitro is an important limiting factor in this respect. Although many aspects, including culture environment, restricted time in culture, off-target differentiation, and limited corticomedullary organization play a role in restricting maturation, the absence of a functional vasculature is of particular importance. In embryology, the development of the kidney depends on the presence of perfused blood vessels. These supply the tissue with oxygen and nutrients and enable the interaction between podocytes and vascular endothelial cells (ECs) that is necessary for their maturation and development of the glomerular basement membrane (GBM)^5–7^. In kidney organoids maintained in vitro, this interaction does not occur, limiting their maturation as well as their resemblance to in vivo kidney tissue.

We and others have shown previously that transplantation in mice leads to vascularization and progressive maturation of hiPSC-derived kidney organoids^8–10^, which even enables functional glomerular filtration^11^. However, this model is labor intensive and unsuitable to study large numbers of organoids. Transplantation on the chicken chorioallantoic membrane (CAM) is much more straightforward and was demonstrated to induce vascularization^12^. Because the CAM takes time to develop, this method allowed for only 5 days of transplantation, limiting the extent of the vasculature and maturation. Therefore, we aimed to develop an easy, efficient, reproducible and accessible model to induce and study vascularization and maturation of kidney organoids through transplantation in the coelomic cavity of chicken embryos. Chicken embryos can develop normally after opening the egg shell, lack a fully functional immune system, and their coelomic cavity is a favorable environment for vascularization and differentiation of transplanted embryonic tissues^13–17^. Unlike grafting on the CAM, the coelom permits transplantation as early as day 3–4 of embryonic development, allowing for a longer duration of transplantation, and quite unlimited expansion of the transplanted tissue in all directions^16^.

Here, we transplanted hiPSC-derived kidney organoids inside the coelom of chicken embryos and extensively investigated the effect of transplantation on the vasculature, cellular composition and maturation of the organoids using immunofluorescence (IF), single cell RNA sequencing (scRNAseq), and ultra-large scale transmission electron microscopy (TEM)^18^. To enable analysis of the 3D organization of organoid glomeruli, we performed serial block face scanning electron microscopy (SBF-SEM) followed by artificial intelligence-based segmentation, annotation and 3D reconstruction. We demonstrate the development of a chimeric perfused vascular network inside transplanted organoids, which invades the glomerular structures and induces enhanced nephron maturation as well as improved stromal composition.

## Results

### Kidney organoids are vascularized upon intracoelomic transplantation in chicken embryos

**Fig. 1:**
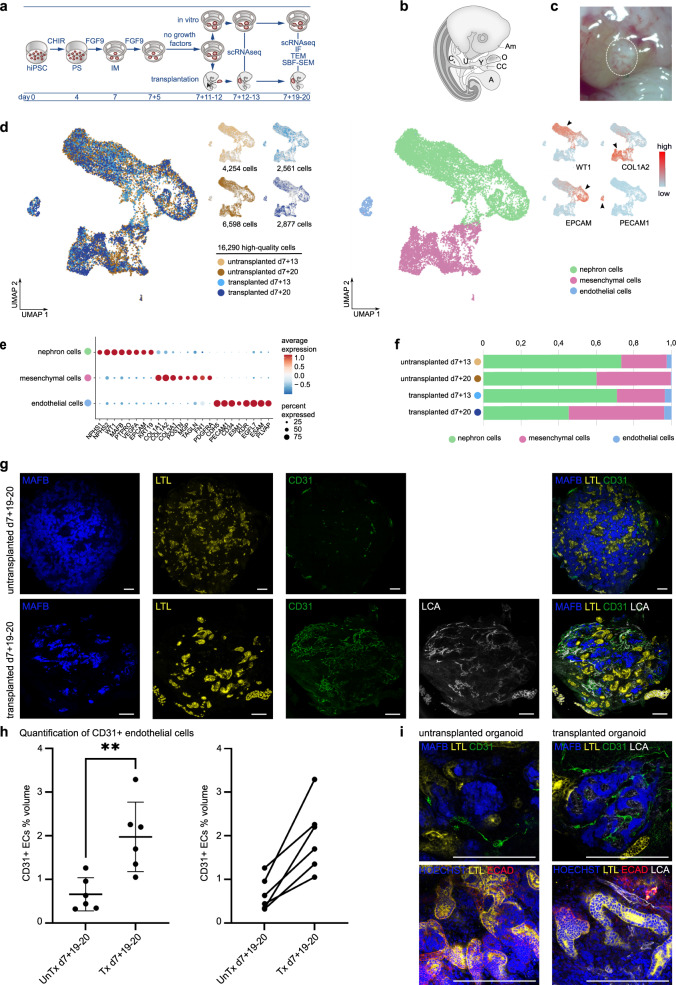
hiPSC-derived kidney organoids become vascularized upon transplantation in the coelomic cavity of chicken embryos. **a** Schematic of the in vitro generation of kidney organoids and transplantation inside chicken embryos. HiPSCs were differentiated for 7 days as monolayer, followed by culture as organoids on an air liquid interface. On d7+11 or d7+12 of differentiation, they were either transplanted inside the coelomic cavity of HH23 chicken embryos or maintained in vitro. Transplanted organoids and untransplanted controls were harvested for analysis with scRNAseq 1 day after transplantation and for scRNAseq, IF, TEM, and SBF-SEM 8 days after transplantation. **b** Schematic of the method of transplantation inside the coelomic cavity of chicken embryos *(modified from Dossel et al*. *Science 1954, with permission*)^19^: a small hole is made in the chorion and amnion membrane using forceps, the bisected organoid is inserted into the coelom with a blunt instrument through this hole and the opening in the body wall that is still present at this stage of development. *A* allantois*, Am* amnion, *C* coelom, *CC* cut chorion membrane, *O* organoid, *U* umbilical ring, *Y* yolk stalk. **c** Macroscopic image of a vascularized kidney organoid (circled area) attached to the liver inside a day 12 chicken embryo. **d** UMAP visualization of a total of 16,290 high-quality human organoid cells obtained from untransplanted (4254 cells from d7+13, 6598 from d7+20) and transplanted (2561 cells from d7+13, 2877 from d7+20) kidney organoids, color-coded by condition (left) or main cell populations: nephron cells, mesenchymal cells and endothelial cells (right). Top right inset: UMAP plot color-coded by the expression level of *WT1*, *COL1A2*, *EPCAM,* and *PECAM1* genes (red: high expression level, blue: low expression level). **e** Dot plot representing marker gene expression in organoid cell clusters. Dot size indicates proportion of cells in cluster expressing a gene, color intensity indicates the level of expression. **f** Relative cluster quantification for each condition, showing endothelial cell loss upon prolonged culture in vitro and maintenance upon transplantation. The proportion of nephron cells decreased in favor of mesenchymal cells in d7+20 compared to d7+13 organoids, with the highest proportion of mesenchymal cells in transplanted d7+20 organoids. Source data are provided as a source data file. **g** Immunofluorescent images of untransplanted (top) and transplanted (bottom) kidney organoids, showing glomerular (MAFB-BFP2 (blue)) and tubular (LTL (yellow)) structures and endothelial cells (CD31 (green)). In transplanted organoids the endothelial cells have formed a vascular network and have become perfused as demonstrated by the presence of injected LCA (white). Scale bars 200 µm. **h** Quantification of CD31 positive ECs as percent volume of organoid volume in whole mount untransplanted (untx) and transplanted (tx) organoids at d7+19–20. *n* = 6 untransplanted and six matched transplanted organoids from six different experiments. Left: Transplanted organoids contain significantly more EC volume than untransplanted controls (*p* < 0.01). Data are presented as individual data points, mean (SD). Unpaired two-tailed t-test was performed to compare means between group. Right: Alternative visualization demonstrating the increase in EC percent volume upon transplantation for each differentiation. Source data are provided as a source data file. **i** Top: Glomerular structures (MAFB-BFP2 (blue)) in untransplanted kidney organoids did not contain human endothelial cells (CD31 (green)), while these structures were invaded by endothelial cells (CD31 (green), LCA (white)) upon transplantation. Bottom: After incubation inside the chicken embryo, tubular structures (LTL (yellow) and ECAD (red)) were aligned by perfused capillaries (LCA (white)). Scale bars 200 µm. Images in **g**, **i** are based on >5 separate experiments and 3 cell lines (iPSC-MAFB (shown here), LUMC0072 (Supplementary Fig. 3), LUMC0020 (Supplementary Fig. 3)).

Chicken embryos are easily accessible, require very little maintenance, and their coelomic cavity is a favorable environment for the differentiation of embryonic tissues^15–17,19^. Therefore, we aimed to investigate the suitability of intracoelomic transplantation to induce and study vascularization and maturation of hiPSC-derived kidney organoids. Kidney organoids were generated from hiPSCs using previously published protocols (Fig. 1a)^10,20^. Although organoids generated in this manner contain some ECs, these fail to invade glomerular structures and diminish upon prolonged in vitro culture (Supplementary Fig. 1A)^21^. To ensure the presence of endogenous ECs at the time of transplantation, we decided to transplant organoids at a relatively early stage, on d7+11 or d7+12 of differentiation. Organoids were bisected and inserted into the coelomic cavity of day 4 (Hamburger Hamilton stage 23) chicken embryos (Fig. 1a, b)^19,22^. At this stage of development, the coelom is large enough to accommodate half an organoid, and the body wall is not yet fully closed, allowing straightforward transplantation that takes around 5 min per embryo. Transplanted organoids and untransplanted controls were harvested 1 day after transplantation for scRNAseq analysis and 8 days after transplantation for scRNAseq, IF, TEM and SBF-SEM analysis (Fig. 1a). Embryos selected for IF analysis were injected with Rhodamine labeled *lens culinaris agglutinin* (LCA)^23^ to stain the endothelium of perfused blood vessels before sacrificing them. Organoids had most frequently become attached to the liver, but could also be found connected to the intestines or ribs. Upon visual inspection through a stereo microscope, they appeared to be vascularized with apparent blood perfusion (Fig. 1c).

For scRNAseq analysis, we performed enzymatic dissociation of 94 untransplanted and 75 bisected transplanted organoids (150 chicken embryos) at d7+13 and 93 untransplanted and 46,5 bisected transplanted organoids (93 chicken embryos) at d7+20 from the same differentiation to single cells. Pooling such a high number of organoids for each condition allows for mitigating potential inter-organoid variability. An aliquot was taken from the single cell suspension of each condition and processed for sequencing. After species demultiplexing (for transplanted organoids), quality controls and filtering, a total of 16,290 high quality human organoid cells and 6866 high quality chicken host cells were retained for downstream analyses (Fig. 1d, Supplementary Figs. 2A, 3A, and Supplementary Data 1). Unsupervised clustering of the organoid-derived human cells revealed three main cell populations, which were identified as nephron, mesenchymal and endothelial cells based on expression of established marker genes (Fig. 1d, e and Supplementary Data 2). The proportion of nephron cell types was the highest at d7+13 (73% of the total cell number in untransplanted and 71% in transplanted organoids) and had decreased in favor of mesenchymal cell types at d7+20 (60% in untransplanted, 45% in transplanted). As expected, in organoids cultured in vitro the proportion of ECs decreased over time from 2.6% at d7+13 to 0.4% at d7+20. Upon transplantation, however, we observed an increase to 4% of ECs at d7+20 (Fig. 1f and Supplementary Data 3). Whole mount IF staining of untransplanted and transplanted organoids at d7+19–20 followed by quantification of organoid derived ECs (human CD31+) confirmed the higher percentage of ECs in transplanted organoids (Fig. 1g, h). The presence of injected rhodamine-labeled LCA demonstrated perfusion of the vasculature (Fig. 1g and Supplementary Fig. 1B). All investigated transplanted organoids had become vascularized. In addition, the perfused blood vessels (CD31+, LCA+) were shown to invade the organoid glomerular structures marked by an mTagBFP2 podocyte reporter (MAFB+) or stained for nephrin (NPHS1+) and align with tubular structures (LTL+/ ECAD+) (Fig. 1I and Supplementary Fig. 1B). Host-derived ECs also appear to contribute to the vasculature, as evidenced by the presence of CD31−, LCA+ (perfused chicken derived) vessels beside CD31+, LCA+ (perfused organoid derived) and CD31+, LCA− (unperfused organoid derived) vessels (Supplementary Fig. 4A). Unperfused chicken-derived endothelial cells could not be identified, because a specific antibody for these cells is not available. Within the chicken cells collected with transplanted organoids for scRNAseq, an angiogenic EC population was identified (Supplementary Fig. 3B–D and Supplementary Data 4). Some of these might reflect host-derived ECs that invaded the kidney organoids upon transplantation in addition to the endogenous human ECs. However, liver-specific sinusoidal ECs, as well as hepatocytes and other non-renal cell types were also identified, likely emerging from chicken tissue that remained attached to the kidney organoids during collection despite careful dissection, resulting in challenging interpretation of the chicken cell dataset (Supplementary Fig. 3B–D).

Since vascularization was already quite extensive after only 8 days of transplantation, we investigated the development of the vasculature in the organoids over time. On day 1 after transplantation, perfused blood vessels (LCA+) could already be detected at the edge of the transplanted organoids (Supplementary Fig. 4B). Three days after transplantation, the first glomerular-like structures (MAFB+) started to be invaded by human ECs (CD31+/LCA+). On day 5, vascularized glomerular structures could be seen throughout the organoid, and on day 7, the majority of glomerular structures were perfused (Supplementary Fig. 4B).

### Heterogeneity of nephron cell types in kidney organoids is preserved upon transplantation

**Fig. 2:**
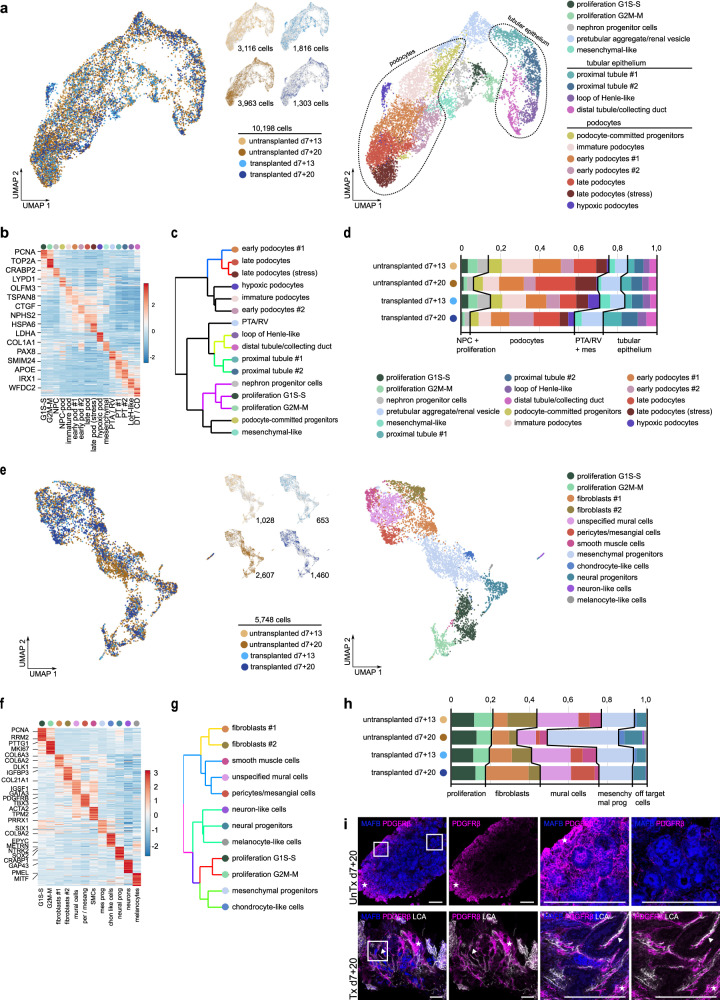
Heterogeneity of nephron and mesenchymal cell types in untransplanted and transplanted kidney organoids. **a** UMAP visualization of a total of 10,198 high-quality nephron cells obtained from untransplanted (3116 cells from d7+13, 3963 from d7+20) and transplanted (1816 cells from d7+13, 1303 from d7+20) kidney organoids, color-coded by condition (left) and by cluster (*n* = 16; right). **b** Expression-level scaled heatmap of the top 20 marker genes in nephron subclusters. Scale: z-score of the gene expression level. Abbreviations: G1S-S: proliferative nephron cells G1S-S, G2M-M: proliferative nephron cells G2M-M, NPC: nephron progenitor cells, NPC-pod: podocyte-committed progenitors, immature pod; immature podocytes, early pod #1: early podocytes #1, early pod #2: early podocytes #2, late pod: late podocytes, late pod (stress): late podocytes (stress-induced), hypoxic pod: hypoxic podocytes, mesenchymal: mesenchymal-like nephron cells, PTA/RV: pretubular aggregate/renal vesicle, PT #1: proximal tubules #1, PT #2: proximal tubules #2, LoH-like: loop of Henle-like, DT/CD: distal tubules/collecting duct. **c** Hierarchical clustering of nephron cell types, color-coded according to *P*-value from multiscale bootstrap resampling analysis on all highly variable genes. **d** Relative cluster quantification of the nephron cell types in transplanted versus untransplanted kidney organoids (d7+13 and d7+20). Source data are provided as a source data file. **e** UMAP visualization of a total of 5748 high-quality mesenchymal cells obtained from untransplanted (1028 cells from d7+13, 2607 from d7+20) and transplanted (653 cells from d7+13, 1460 from d7+20) kidney organoids, color-coded by condition (left) and by cluster (*n* = 12; right). **f** Expression-level scaled heatmap of the top 20 marker genes in mesenchymal subclusters. Scale: z-score of the gene expression level. Abbreviations: G1S-S: proliferative mesenchymal cells G1S-S, G2M-M: proliferative mesenchymal cells G2M-M, mural cells: unspecified mural cells, per/mesang: pericytes/mesangial cells, SMCs: smooth muscle cells, mes prog: mesenchymal progenitors, chon like cells: chondrocyte-like cells, neural prog: neural progenitors. **g** Hierarchical clustering of mesenchymal cell types, color-coded according to *P*-value from multiscale bootstrap resampling analysis on all highly variable genes. **h** Relative cluster quantification of the mesenchymal cell types in transplanted versus untransplanted kidney organoids (d7+13 and d7+20). The color legend for this figure is the same as the one in (**e**). Source data are provided as a source data file. **i** Staining of PDGFRβ + stromal cells in untransplanted and transplanted kidney organoids reveals abundant PDGFRβ + stromal cells in the periphery of untransplanted organoids (asterix and first magnification) and scarce PDGFRβ + cells in the center (second magnification). Transplantation induces the appearance of perivascular stromal cells (PDGFRβ+, arrowhead and magnifications) supporting perfused blood vessel (LCA+). Magnifications of boxed areas are shown. Scale bars 200 µm.

Next, we evaluated the effect of transplantation and vascularization on the cellular composition of kidney organoids, starting with the nephron compartment. Unsupervised subclustering of a total of 10,198 high quality nephron cells obtained from untransplanted (3116 cells from d7+13; 3963 cells from d7+20) and transplanted (1816 cells from d7+13; 1303 cells from d7+20) organoids resulted in 16 subclusters, which we identified based on the top marker genes for each cluster (Fig. 2a, b, Supplementary Fig. 2b–d, Supplementary Data 5, 6). Organoids from all conditions contained proliferative cell clusters, nephron precursors (pretubular aggregate/renal vesicle and nephron progenitor cells), tubular cells and podocytes (Fig. 2a). Supporting our cluster identification, further unsupervised hierarchical clustering of the delineated nephron cell phenotypes revealed higher similarity of the different identified podocyte clusters as compared with nephron precursors and proliferating nephron cells, as well as a greater distance with tubular cell clusters (Fig. 2c). More specifically, 4 subclusters of tubular cells were distinguished: 2 types of proximal tubule cells (#1 and 2), loop of Henle-like cells, and distal tubule/collecting duct-like cells (Fig. 2b, c, Supplementary Fig. 2c, and Supplementary Data 5, 7). We did not identify a clear distal convoluted tubule (*SLC12A3*) or pure collecting duct (*AQP2/4*) population. The podocytes displayed more heterogeneity, resulting in 7 subclusters, mainly reflecting different levels of maturation. The podocyte-committed progenitor cluster highly expressed *OLFM3*, a podocyte-specific lineage marker, but no other markers for podocyte identity. The immature podocyte, early podocyte #1 and 2 and late podocyte clusters expressed increasing levels of markers for podocyte identity (*MAFB*, *WT1*), and differentiation (*NPHS1* and *2*, *PODXL*, *CLIC5*) (Fig. 2b, Supplementary Fig. 2b, and Supplementary Data 5). In addition, we found a subcluster of late podocytes expressing genes associated with stress and a subcluster of hypoxic podocytes (Fig. 2b and Supplementary Fig. 2b, e). Since all organoids from the same condition were pooled, it is not possible to definitively distinguish whether these podocyte subclusters are the result of different maturation stages within the same or between different organoids. However, based on variation observed through TEM imaging, we believe it reflects intra- rather than inter-organoid variability.

All nephron subclusters were represented in all organoid conditions, and major shifts in populations were not observed (Fig. 2d). However, there were some interesting differences between conditions. In transplanted organoids (d7+13 and d7+20), the proportion of hypoxic podocytes appeared increased, which can be explained by the hypoxia in the intracoelomic cavity prior to vascularization. This did not lead to a larger proportion of stress-induced podocytes, which were most abundant in untransplanted d7 + 20 organoids.

### Transplantation induces an increase in perivascular cell types

Mesenchymal cells are essential for kidney development and function. However, they can also contribute to kidney fibrosis, and subpopulations of stromal cells have been reported in organoids to give rise to off-target cell populations such as cartilage^24^. To investigate the identity of the mesenchymal cells in our kidney organoids, we performed unsupervised subclustering of the 5748 high quality mesenchymal cells obtained from untransplanted (1028 cells from d7+13; 2607 cells from d7+20) and transplanted (653 cells from d7+13; 1460 cells from d7+20) organoids (Fig. 2e). This revealed 12 clusters, which we identified as proliferating cells, fibroblasts, mural cells, mesenchymal progenitor cells and off-target cell populations based on their top marker gene expression (Fig. 2e, f and Supplementary Data 8). Further unsupervised hierarchical clustering supported our cluster identification with grouping clusters by cell types: fibroblasts, mural cells, off-target cells derived from neural lineage, and proliferating cells together with mesenchymal progenitors and derivative off-target chondrocyte-like cells (Fig. 2g). More specifically, off-target cells consisted of chondrocyte-like cells, neural progenitors, neuron-like cells and melanocyte-like cells. Three types of mural cells, namely pericytes/mesangial cells, smooth muscle cells, and unspecified mural cells could be distinguished (Fig. 2e, f and Supplementary Fig. 2f). In untransplanted organoids at d7+20, the proportion of off-target cell types and mesenchymal progenitor cells was increased compared to d7+13, at the expense of mural cells. In transplanted organoids at d7+20, the increase in off-target cell types and mesenchymal progenitors was much less pronounced, but we did observe an increase in pericytes/mesangial cells and fibroblasts (Fig. 2h and Supplementary Data 3). We confirmed the presence of perivascular stromal cells in transplanted organoids through IF staining with a PDGFRβ antibody, combined with injected rhodamine-labeled LCA. In untransplanted organoids, PDGFRβ positive stromal cells were present mainly in the periphery of the organoid and only sporadically in the center scattered between the epithelial structures. In transplanted organoids, perivascular PDGFRβ+ stromal cells supporting the LCA+ perfused blood vessels had appeared, although some areas with PDGFRβ+ stromal cells were still visible between epithelial structures (Fig. 2I).

These data imply that, although transplantation in chicken embryos may increase the proportion of mesenchymal cells, it favors their differentiation to ‘on-target’ pericytes and mesangial cells, while reducing the amount of off-target cell types compared to in vitro controls.

### Vessel wall stabilization in transplanted organoids

**Fig. 3:**
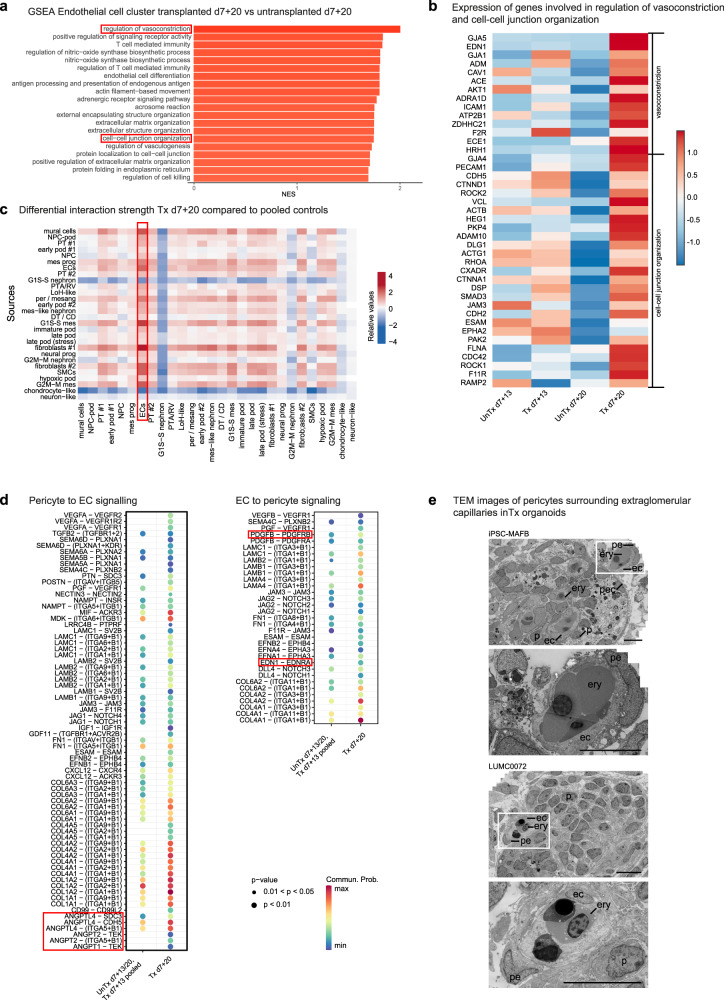
Vessel wall stabilization in transplanted kidney organoids. **a** Gene set enrichment analysis (GSEA) for EC cluster in transplanted d7+20 versus untransplanted d7+20 organoids demonstrating enrichment of gene sets associated with vasoconstriction and cell–cell junction organization. NES: normalized enrichment score. Source data are provided as a source data file. **b** Expression-level scaled heatmap of genes involved in vasoconstriction and cell–cell junction organization, demonstrating upregulation of the majority of these genes in d7+20 transplanted endothelial cells compared to all other conditions. Scale: z-score of the gene expression level. **c** Heatmap demonstrating differential interaction strength (probability of interactions) per cluster for d7+20 transplanted organoids compared to pooled controls (d7+13 transplanted and untransplanted and d7+20 untransplanted organoids). Abbreviations: mural cells: unspecified mural cells, NPC-pod: podocyte-committed progenitors, PT #1: proximal tubules #1, early pod #1: early podocytes #1, NPC: nephron progenitor cells, mes prog: mesenchymal progenitors, ECs: endothelial cells, PT #2: proximal tubules #2, G1S-S nephron: proliferative nephron cells G1S-S, PTA/RV: pretubular aggregate/renal vesicle, LoH-like: loop of Henle-like, per/mesang: pericytes/mesangial cells, early pod #2: early podocytes #2, mes-like nephron: mesenchymal-like nephron cells, DT/CD: distal tubules/collecting duct, G1S-S mes: proliferative mesenchymal cells G1S-S, imm pod; immature podocytes, late pod: late podocytes, late pod (stress): late podocytes (stress-induced), fibroblasts #1: fibroblasts #1, neural prog: neural progenitors, G2M-M nephron: proliferative nephron cells G2M-M, fibroblasts #2; fibroblasts #2, SMC: smooth muscle cells, hypoxic pod: hypoxic podocytes, G2M-M mes: proliferative mesenchymal cells G2M-M, chondrocyte-like: chondrocyte-like cells, neuron-like: neuron-like cells. **d** Dot plot of the ligand-receptor pairs for which interaction strength between pericytes and endothelial cells is increased in d7+20 transplanted organoids compared to pooled controls with (left) pericytes as source and (right) endothelial cells as source. **e** In transplanted kidney organoids, extraglomerular capillaries are supported by pericytes. *ec* endothelial cell, *p* podocyte, *pe* pericyte, *pec* parietal epithelial cell, *ery* erythrocyte. Scale bars 10 µm.

Stabilization and maturation of newly formed blood vessels requires the establishment of inter-EC contacts and pericyte-EC interactions^25,26^. We evaluated the occurrence of these events in our model through gene set enrichment analysis (GSEA) of ECs in d7+20 transplanted compared with respective untransplanted organoids and ligand-receptor (LR) network analysis. Differential gene expression analysis (DGEA) followed by GSEA in ECs demonstrated upregulation of, amongst others, gene sets associated with vascularization, regulation of vasomotor tone, immunity (likely due to the connection to the chicken embryo circulation), and extracellular matrix organization (occurs in but is not specific for angiogenesis and vessel stabilization). Interestingly, a gene set associated with cell–cell junction organization, an important event in vessel wall stabilization, as well as a gene set involved in the regulation of vasoconstriction, reflecting maturation of the vasculature were significantly enriched (Fig. 3a and Supplementary Data 9). Indeed, expression of many individual genes involved in these processes were upregulated in transplanted kidney organoids at d7+20 compared to all other conditions (Fig. 3b). To investigate the interaction of ECs with pericytes, we performed LR network analysis. Because of the low number of both pericytes/mesangial cells and ECs, especially in untransplanted d7+20 organoids, we decided to pool the cells from this condition with those from untransplanted and transplanted d7+13 organoids, and compare them to transplanted d7+20 organoids for further LR analysis. Since the observed increase in proportion and change in localization of pericytes was clearly present in transplanted d7+20 organoids compared to the other conditions, changes in interactions between ECs and pericytes upon transplantation were expected to remain detectable after pooling of cells from these 3 conditions. Investigation of the differential interaction strength (reflecting the probability of interactions) for all clusters revealed an increase of inferred interactions between ECs and mesenchymal cell types in transplanted d7+20 organoids compared to pooled controls, especially with the ECs as receiver (Fig. 3c). We subsequently analyzed which ligand-receptor interactions were newly detected or increased from pericytes to ECs and vice versa (Fig. 3d). We observed an increase in a large number of LR-pairs, the majority of which have been reported to play a role in angiogenesis (SEMA-PLXN^27^, PTN-SDC3^28^, EFN-EPH^29–32^, IGF1-IGF1R, GDF11-TGFBR1 + ACVR2B^33^), vascular development (CXCL12-CXCR^34^, FN1-ITGA^35^), pericyte recruitment to neovasculature (PDGFB-PDGFRB, EDN1-EDNRA), vessel wall stabilization (ANGPT-TEK, JAG/DLL4-NOTCH^36,37^), or generally in pericyte-endothelial cell interaction (TGFB2-TGFBR^38^, VEGF-VEGFR^39^, ESAM-ESAM^40^). For the two most relevant identified upregulated pathways, *undefined* ANGPT-TEK and PDGF-PDGFR, we generated heatmaps to visualize the expression of the genes encoding the ligands and receptors in all organoid cell clusters (Supplementary Fig. 5a, b). *ANGPT1* was expressed by pericytes and unspecified mural cells, but also by podocytes, which is in accordance with the reported expression in kidney development^41^. *ANGPT2* was expressed by endothelial cells and mural cells. As expected, all receptors for angiopoietins were most highly expressed in endothelial cells. *PDGFB* was strongly expressed by endothelial cells and its receptor *PDGFRB* in mural cells and to a lesser degree in other mesenchymal cell types and several subsets of podocytes.

Inferred interactions with collagens and laminins were broadly upregulated in transplanted organoids, which can play a role in but is not specific for vessel maturation. Also, several upregulated LR-pairs have not been described previously in physiological vascularization, namely *POSTN*-*ITGAV* + *ITGB5* (*POSTN* implicated in kidney fibrosis), *PGF*-*VEGFR1* (mainly described in pathological angiogenesis), *CD99*-*CD99L2* and *MIF*-*ACKR3* (involved in neutrophil extravasation and attraction), *JAM3*-*JAM3* and *NECTIN3*-*NECTIN2* (part of endothelial cell tight and adherens junctions) and *LRRC4B*-*PTPRF* (mainly described in neurons). These could be examples of unwanted effects of transplantation or previously unrecognized physiological interactions. Distinguishing between these would require further investigation.

Finally, to add spatial information to these sequencing data, we performed TEM to evaluate the presence of perivascular stromal cells in transplanted organoids, and identified pericyte-like cells supporting extraglomerular capillaries (Fig. 3e).

### Vascularization improves the maturation of the kidney organoids

**Fig. 4:**
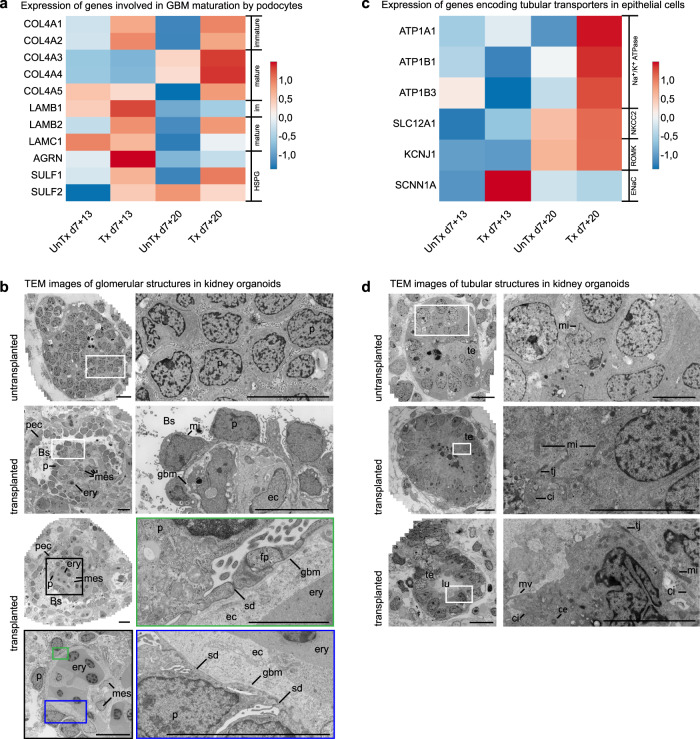
hiPSC-derived kidney organoids mature upon transplantation. **a** Expression-level scaled heatmap of genes involved in GBM development in podocytes in untransplanted versus transplanted kidney organoids at different timepoints. *COL4A3-5* and *LAMB2* are upregulated in d7+20 transplanted podocytes compared to all other conditions, fitting with the switch from collagen IV α1α2α1 to collagen IV α3α4α5 and from laminin β1 to laminin β2 during GBM maturation. *LAMC1* and *AGRN*, encoding laminin γ1 and agrin (a heparan sulfate proteoglycan HSPG) in the mature GBM are not upregulated in d7+20 transplanted podocytes, implying the GBM in the organoids is not fully matured. Scale: z-score of the gene expression level. **b** Transmission Electron Microscopy (TEM) imaging displays maturation of glomerular structures upon transplantation for 8 days. Podocyte clusters are invaded with capillaries containing erythrocytes and supported by mesangial cells. Parietal epithelial cells form a Bowman’s capsule, a glomerular basement membrane is deposited between the podocytes and endothelial cells, and rudimentary slit diaphragms are formed between primitive foot processes. Magnifications of the boxed areas are displayed, *Bs* Bowman’s space*, ec* endothelial cell*, ery* erythrocyte, *fp* foot process*, gbm* glomerular basement membrane, *leu* leukocyte, *mes* mesangial cell, *mi* mitochondrion, *p* podocyte*, pec* parietal epithelial cell*, sd* slit diaphragm. Scale bars 10 µm. Scale bar magnification marked in green 2 µm. **c** Expression-level scaled heatmap of genes encoding tubular transporters in untransplanted versus transplanted kidney organoids at different timepoints. Expression of genes encoding the transporters Na^+^/K^+^ ATPase, NKCC2 and ROMK are upregulated in transplanted organoids at d7+20. *SCNN1A*, which encodes the α-subunit of the ENaC channel, is not upregulated. Scale: z-score of the gene expression level. **d** Upon transplantation for 8 days, tubular structures also show signs of maturation. Tubular epithelial cells have formed a monolayer and their nuclei have moved toward the basolateral side of the cell. Tight junctions, cilia, a centriole, microvilli and abundant mitochondria are visible. *ce* centriole, *ci* cilium, *lu* lumen, *mi* mitochondrion, *mv* microvilli, *te* tubular epithelium, *tj* tight junction. Scale bars images left row 10 µm, scale bars magnifications right row 5 µm. TEM Images based on 5 transplanted organoids from 2 experiments using 2 cell lines (MAFB (here)) and LUMC0072 (Supplementary Fig. 1C, D).

Cell-cell communication is essential for kidney development and maintenance. Interestingly, a comparison of the total number of interactions and interaction strength of all cell clusters between conditions showed a marked decrease in interactions in untransplanted d7+20 organoids compared to all other conditions, suggesting overall loss of cell-cell communication in in vitro culture (Supplementary Fig. 2G). To further evaluate the effect of transplantation and vascularization on nephron cell types, we compared late stage transplanted organoids with respective untransplanted controls through DGEA followed by GSEA. This analysis was performed for the late podocyte cluster and tubular epithelial cells (proximal, loop of Henle and distal clusters combined). In transplanted late podocytes, we observed enrichment of gene sets associated with VEGF production, vasculogenesis, organization of cell–cell junctions, and establishment and maintenance of cell polarity (Supplementary Fig. 6A and Supplementary Data 10). This is consistent with the observed vascularization and indicates differentiation of podocytes from immature simple columnar epithelium to a more mature phenotype displaying apicobasal polarity and forming intercellular junctions at their basal side. Interestingly, a gene set associated with positive regulation of collagen biosynthetic processes was also enriched in transplanted podocytes. We hypothesized that this was related to the establishment of a GBM, which in embryonic development is formed by the fusion of two basement membranes produced by both podocytes and ECs. Therefore, we investigated the expression of genes encoding specific collagens, laminins and heparan sulfate proteoglycans (HSPGs) involved in the formation and maturation of the GBM by podocytes in untransplanted and transplanted organoids (Fig. 4a). We observed upregulation of *COL4A3–5* and *LAMB2* in d7+20 transplanted podocytes compared to all other conditions, fitting with the switch from collagen IV α1α2α1 to collagen IV α3α4α5 and from laminin β1 to laminin β2 during GBM maturation. Also, *SULF1*, a regulator of heparan sulfates, is highly expressed in d7+20 transplanted podocytes. *LAMC1* and *AGRN*, encoding laminin γ1 and agrin, a dominant laminin and HSPG in the mature GBM, and *SULF2* are not upregulated in d7+20 transplanted podocytes, implying the GBM in the organoids is not fully matured.

To assess whether glomerular maturation and GBM formation were also detectable at an ultrastructural level, we performed TEM imaging followed by stitching (Fig. 4b and Supplementary Fig. 1C). In contrast to traditional TEM, in which only small areas selected based on low-resolution overviews are imaged at higher magnification, this yields large 2D high-resolution virtual slides^18^. After 8 days of transplantation, the invasion of organoid glomerular structures by capillaries containing chicken-derived nucleated erythrocytes and leukocytes had resulted in the rearrangement of podocytes around the capillaries and the deposition of a GBM between the podocytes and ECs. Podocyte cell bodies stretched out along the GBM, cell junctions between adjacent podocytes moved to the basal side of the cells, and primitive foot processes were formed. Rudimentary slit diaphragms were observed between these primitive foot processes. ECs were variable in appearance but generally contained large nuclei surrounded by relatively thick cell bodies, lacking the fenestrations typical for mature human glomeruli (Fig. 4b). Mesangial cells were visible in the stalk of some of the glomeruli, adjacent to the ECs (Fig. 4b). The vascularized glomerular structures were surrounded by a single layer of parietal epithelial-like cells and a clearly distinguishable Bowman’s space had appeared between the podocytes and parietal epithelial-like cells (Fig. 4b).

The GSEA for d7+20 transplanted tubular epithelial cells versus untransplanted controls demonstrated enrichment of gene sets associated with establishment and maintenance of cell polarity and of potassium ion transport (Supplementary Fig. 6B and Supplementary Data 11). Since the development of transporters is indispensable for tubular functionality, we evaluated the expression of genes encoding several major tubular transporters in untransplanted and transplanted organoids. We observed upregulation of genes encoding the sodium-potassium pump Na^+^/K^+^-ATP-ase (*ATP1A1, ATP1B1*, *and* *ATP1B3*), the sodium potassium chloride cotransporter NKCC2 (*SLC12A1*) and the renal outer medullary potassium channel ROMK (*KCNJ1*) in d7+20 transplanted organoids compared to the other conditions (Fig. 4c). The expression of *SCNN1A*, which encodes the α-subunit of the epithelial sodium channel (ENaC) in the distal convoluted tubule, connecting tubule and collecting duct, was surprisingly highest in transplanted d7+13 organoids (Fig. 4c). Expression of *SLC12A3* and *AQP2*, encoding the sodium chloride cotransporter (NCC) in the distal convoluted tubule and the water channel aquaporin 2 in the collecting duct, was not detected in any condition, consistent with the lack of clearly distinct distal convoluted tubule and collecting duct cell clusters (Fig. 2a–c). TEM analysis of tubular structures demonstrated that the lumen widened upon transplantation, the epithelial cells formed a monolayer and, in accordance with the GSEA, showed signs of apicobasal polarity with their nuclei moving towards the basolateral membrane. Abundant mitochondria were observed in their cytoplasm, and some of the tubular cells displayed cilia and microvilli, further reflecting ongoing maturation (Fig. 4d and Supplementary Fig. 1D).

### Glomerular morphogenesis to capillary loop stage

**Fig. 5:**
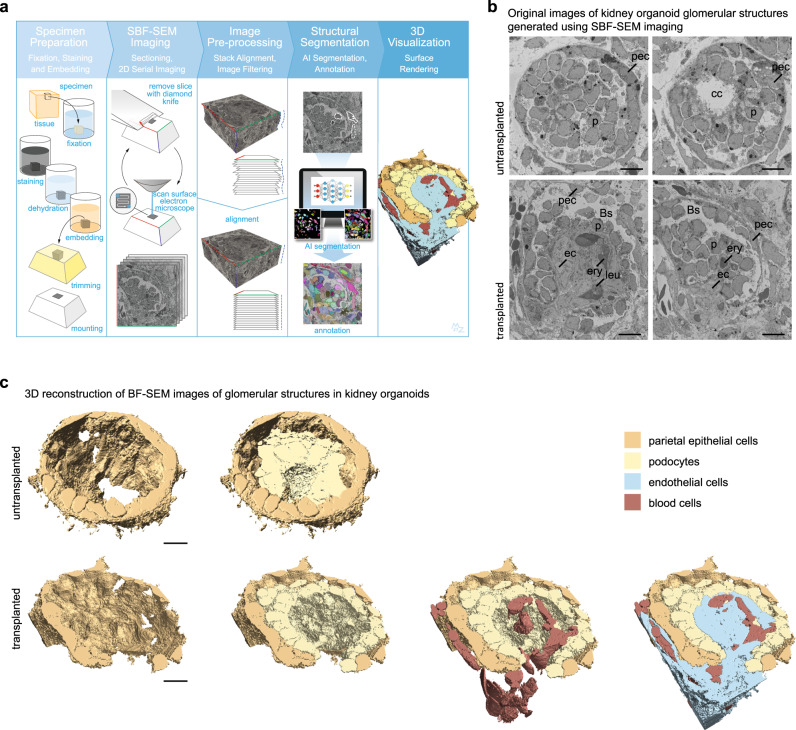
SBF-SEM followed by 3D reconstruction enables analysis of the 3D organization of organoid glomerular structures and reveals maturation to capillary loop stage. **a** The SBF-SEM imaging workflow comprises five steps. First the specimen is prepared for imaging by chemical fixation, staining with heavy metals, dehydration and embedding in resin, after which it is prepared for imaging by trimming the block and mounting for SEM. SBF-SEM imaging is performed inside the microscope and consists of alternating sectioning and imaging steps, by which a stack of images through the specimen block is generated. Image pre-processing is performed by image alignment and filtering. Segmentation of cell structures, such as nucleus, mitochondria and endoplasmic reticulum was performed by Artificial Intelligence methods followed by manual annotation of cell types. Visualization of images and movies is performed by surface rendering of segmented structures. **b** Images generated by SBF-SEM of glomerular structures in untransplanted (top) and transplanted (bottom) kidney organoids (iPSC-MAFB). For full Z-stacks, see Supplementary Movies 1, 2. *Bs* Bowman’s space, *cc* central cavity, *ec* endothelial cell, *ery* erythrocyte, *leu* leukocyte, *p* podocyte, *pec* parietal epithelial cell. Scale bar 10 µm. **c** 3D reconstruction of the SBF-SEM datasets in B and Supplementary Movies 1, 2. In the untransplanted glomerular structure (top) a layer of parietal epithelial-like cells (beige) is visible surrounding a cluster of podocytes (yellow) containing a central cavity. In the transplanted glomerular structure (bottom) a perfused capillary (endothelial cells in blue, blood cells in red) invades the glomerulus and forms a single loop inside the glomerulus. The podocytes (yellow) have reorganized around the capillary and the PECs (beige) have adopted a more flattened phenotype. For 360° view of the 3D reconstructions, see Supplementary Movies 5, 6. Scale bar 10 µm. For SBF-SEM analysis, 2 transplanted organoids and 1 untransplanted organoid, all differentiated from iPSC-MAFB, were used to generate 4 datasets of untransplanted and 3 datasets of transplanted glomerular structures. 2 datasets (1 transplanted and 1 untransplanted) were used for the 3D visualization shown in (**c**).

To evaluate the 3D organization of glomerular cells and structures in untransplanted and transplanted organoids, we performed SBF-SEM. This technique utilizes an ultramicrotome mounted inside the vacuum chamber of a scanning electron microscope to serially image the block face of a tissue sample, of which ultrathin sections are cut after the generation of each image. The result is a Z-stack of images that allows for the evaluation of the 3D tissue structure as well as ultrastructural analysis at a subcellular level^42^. After generating datasets of 4 untransplanted and 3 transplanted glomerular structures (Supplementary Movies 1–4), artificial intelligence-based segmentation and annotation was performed on 2 datasets (1 untransplanted and 1 transplanted) (Supplementary Movies 1, 2), followed by manual annotation of cell types, segmentation and 3D visualization (Fig. 5a and Supplementary Movies 5, 6).

The untransplanted glomerular structure consisted of a cluster of podocytes, surrounded by a layer of parietal epithelial-like cells (Fig. 5b, c and Supplementary Movies 1, 5). After 8 days of transplantation, the glomerular structure had become vascularized. A perfused capillary containing erythrocytes as well as leukocytes was clearly visible running along the side of the glomerular structure, before invading it and forming a single loop reaching deep into the center of the glomerulus before exiting it (Fig. 5b, c, Supplementary Movies 2, 6). The podocytes reorganized into a layer surrounding the capillary and their cell bodies stretched out along the ECs (Fig. 5c). The parietal epithelial-like cells assumed a more flattened morphology and formed a nearly complete capsule around the podocytes (Fig. 5c and Supplementary Movie 6).

## Discussion

In this study, we demonstrate a new model to induce and study vasculogenesis and maturation in hiPSC-derived kidney organoids through transplantation inside the coelomic cavity of chicken embryos. The procedure is easy and efficient, requiring around 5 min per embryo, and provides a scalable alternative to the labor-intensive transplantation in mice. We limited transplantation duration to 8 days in order to sacrifice the embryos on day 12 of incubation, a day before it is believed they start experiencing pain^43,44^. An extension to a maximum of 15 days is theoretically possible, sacrificing the embryos on day 19 of incubation to avoid hatching. Endogenous organoid-derived ECs which diminish in in vitro culture, thrive upon transplantation, invade glomerular structures and form a chimeric vasculature with invading host-derived endothelium. This implies that organoid vascularization occurs through a combination of vasculogenesis (human, organoid-derived vessels) and angiogenesis (chicken-derived vessels), and is consistent with the main accepted theory that kidney vascularization in embryology depends on angiogenic^45–49^ as well as vasculogenic mechanisms^46,50–52^. The ability of organoid-derived endothelium to contribute to the glomerular vasculature after intracoelomic transplantation signifies the absence of unidentified cues for vascularization in in vitro culture conditions. It is well known that the production of VEGF-A by podocytes during embryonic development is essential for recruitment, differentiation and maintenance of glomerular ECs^5,53^. Although it has been demonstrated that organoids in vitro produce VEGF^10^, either the levels produced or the concentration gradient within the organoid could be insufficient. The addition of VEGF to organoid culture media, which increases VEGF level but does not establish a gradient, supports EC proliferation, but fails to induce glomerular vascularization^54^. In our transplanted podocytes, we found upregulation of a gene set associated with VEGF production (Supplementary Fig. 6A), and gene expression of VEGFA was clearly enhanced in podocytes compared to all other organoid cell clusters (Supplementary Fig. 5C) implying that the establishment of a larger VEGF gradient by the podocytes could be a major facilitator of vascularization upon transplantation. Other factors that could play a role are shear stress and hypoxia. Shear stress is an important regulator of EC maturation and establishment of the endothelial glycocalyx^55–57^. The static culture of kidney organoids is therefore likely to be suboptimal for vascular development. Indeed, it has been shown that culturing organoids under flow leads to proliferation of ECs, although invasion of glomerular structures was still rare^58^. However, shear stress in this study was much lower than reported as physiological in embryology^57,59,60^ and modifications were made to the culture conditions, such as addition of fetal bovine serum and ECM, which could also have contributed to the observed changes. In embryology, nephron progenitor cells are thought to reside in a hypoxic environment until they are properly vascularized^61–63^. The cells in our organoids, cultured at an air-liquid interface in an incubator with 20% oxygen, are exposed to much higher oxygen levels. The temporary hypoxic conditions they are exposed to in the coelomic cavity of chicken embryos might therefore mimic physiologic conditions more closely. Unfortunately, the number of ECs in our scRNAseq dataset was too small to evaluate the influence of the conditions discussed above on transplanted ECs.

Analysis of the effect of transplantation on the cellular composition of kidney organoids revealed an increase in proportion of mesenchymal cells in d7+20 compared to d7+13 organoids, with the highest proportion in transplanted d7+20 organoids. As concerns have been raised about the proliferation of off-target cell types upon transplantation^64^, we analyzed the identity of the mesenchymal cells in our organoids. Interestingly, we found a lower percentage of off-target cells and a higher proportion of pericytes/mesangial cells and fibroblasts in transplanted organoids at d7+20 compared to untransplanted controls. This is consistent with the reduction of off-target cell types previously reported upon transplantation in mice^24^. The relatively short duration of transplantation in this paper (14 days) and our model (8 days) may partly explain the lack of proliferation of off-target cell types, but not the observed difference with timepoint-matched in vitro controls. The increase in the pericyte/mesangial cell population upon transplantation is an interesting finding. In the human kidney, pericytes play a role in angiogenesis, blood vessel stabilization and blood pressure regulation and have been implied as a source of mesenchymal stem cells^65^. Mesangial cells are specialized pericytes that interact with glomerular ECs and podocytes to enable glomerular functionality^66^. Through LR network analysis and TEM, we were able to demonstrate that the chimeric neovasculature in transplanted organoids is supported by perivascular stromal cells that interact with the ECs to stabilize and mature the vascular network.

Although the duration of transplantation in our study is relatively short compared to that reported in mouse studies, we observed significant maturation of transplanted organoids. Comparison of our scRNAseq data to different transplantation methods would be very valuable. However, only one dataset of kidney organoids transplanted in mice is currently publicly available^24^. Differences in cell line, differentiation protocol, duration of transplantation and dissociation method between this and our study prevent a fair comparison. Hence, further studies, including direct comparison of kidney organoids submitted to different transplantation methods, would be required to investigate this.

GSEA of the late podocyte cluster and tubular epithelial cells revealed upregulation of genes involved in maturation of the GBM and in the development of tubular transporters upon transplantation. Evaluation of the organoids at an ultrastructural level using TEM image mosaics confirmed the deposition of a GBM between the podocytes and the ECs that invaded the glomerular structures. In addition, it demonstrated maturation of the podocytes with the formation of primitive foot processes and rudimentary slit diaphragms. Mesangial cells had followed the ECs into the glomerular structures, providing support to the glomerular vasculature and enabling the interaction with podocytes and ECs that in in vivo kidneys is required for functionality^66^. Tubular epithelial cells in transplanted organoids formed a monolayer, displayed signs of apicobasal polarization, and the tubular lumen widened. Through SBF-SEM followed by 3D reconstruction, we evaluated the spatial organization of organoid glomeruli and visualized the trajectory of the vasculature in a transplanted glomerular structure. A single capillary was visible penetrating deep into the glomerular structure and forming a single loop. It had not yet undergone intussusceptive angiogenesis to form a capillary tuft. The podocytes had reorganized around the capillary.

In conclusion, we have developed a new model to efficiently induce and study the development of a stabilized vascular network in kidney organoids through transplantation in the coelomic cavity of chicken embryos, and demonstrate the essential role of vascularization for organoid maturation and glomerular morphogenesis. This scalable model can be used to identify essential early cues for vasculogenesis and maturation of kidney organoids.

## Methods

### iPSC maintenance and differentiation

hiPSC lines were maintained in Essential 8 medium (E8, Thermo Fisher Scientific) supplemented with 0.5% Penicillin-Streptomycin (Thermo Fisher Scientific) on recombinant human Vitronectin (Thermo Fisher Scientific). All cell lines were mycoplasma free. hiPSCs were passaged twice a week as small clumps using 0.5 mM UltraPure EDTA (Thermo Fisher Scientific). hiPSC reporter MAFB:mTagBFP2 (hiPSC-MAFB)^67^, LUMC0072iCTRL01, and LUMC0020iCTRL6.4 lines (Detailed information can be found at https://hpscreg.eu/) were plated as single cells at 15,000–25,000 cells/cm^2^ one day prior to differentiation using TrypLE Select (Thermo Fisher Scientific) and the addition of RevitaCell Supplement (ThermoFisher Scientific). hiPSCs were incubated from day 0–4 in 8 μM CHIR99021 (R&D Systems) in STEMdiff APEL2 medium (Stem Cell Technologies) supplemented with 1% Protein Free Hybridoma Medium II (PFHMII, Thermo Fisher Scientific) and Antibiotic-Antimycotic solution (Thermo Fisher Scientific). From day 4–day 7 cells were treated with 200 ng mL^−1^ rhFGF9 (R&D Systems) and 1 μg mL^−1^ heparin (Sigma Aldrich) in APEL2-medium. After an 1 h 5 μM CHIR pulse on day 7, cells were dissociated using Trypsin-EDTA (0.25%, Thermo Fisher Scientific), counted and plated on Transwell 0.4 μM pore polyester membranes as pellets of 500,000 cells. These were cultured on an air–liquid interface for another 5 days in APEL2-medium containing 200 ng mL^−1^ rhFGF9 and 1 μg mL^−1^ heparin (only bottom compartment). For the remaining days, the organoids were cultured in medium without FGF9 and heparin, and medium was changed every 2 days. Organoids were maintained on the transwell membranes until transplantation or day 7+20.

### Transplantation in chicken embryos

In accordance with Dutch law, approval by the animal welfare committee was not required for these experiments. Fertilized White Leghorn eggs (*Gallus gallus domesticus*, Drost Loosdrecht B.V.) were placed horizontally in a humidified incubator at 37 °C. After 3 days of incubation, a small hole was made in the tip of each egg by tapping it with the sharp end of a pair of dissecting scissors (Hammacher Karl, HAMMHSB391-10), and 2–3 mL of albumen was removed using a syringe with a 19 gauge needle to lower the embryo inside the egg. A window was then cut into the egg shell using dissecting scissors. 2–3 drops of DPBS with calcium and magnesium (Thermo Fisher Scientific) were added to the egg to avoid dehydration before sealing the window and the hole in the tip of the egg with transparent tape (Tesa 4120). The window was made a day prior to transplantation, because we found that the chorioallantoic membrane, as well as the embryo, have frequently become attached to the egg shell by day 4 of incubation, leading to bleeding and embryo death when attempting to create a window at this timepoint. On day 4 of incubation (Hamburger Hamilton stage 23), kidney organoids on day 7+11 or day 7+12 of differentiation were bisected with a surgical knife and half an organoid was transplanted inside the coelomic cavity of each chicken embryo (Fig. 1a)^22^. Bisection of the organoids was necessary because the coelomic cavity is not large enough to accommodate a whole organoid at this stage of development. Transplantation was performed by creating a small hole in the chorion and amnion membrane with forceps (Hammacher Karl, HAMMHTC090-11), and inserting the bisected organoid through this hole and the opening in the body wall that is still present at this stage of development into the coelom using a blunt instrument (Fig. 1b). The eggs were resealed with transparent tape and further incubated for a maximum of 8 days until the end of the experiment. The whole process of transplantation takes about 5 min per egg, allowing for transplantation in up to 100 chicken embryos per person per day. Since maintenance of the embryos before and after transplantation only requires a humidified incubator, this does not limit the number of embryos used for transplantation. On day 5 and day 12 (respectively 1 and 8 days after transplantation) of incubation, the chicken embryos were sacrificed and the organoids harvested. To enable analysis of the vasculature, some of the embryos were carefully injected with 20 µL of 2.5 mg mL^−1^ rhodamine labeled *lens culinaris agglutinin* (LCA) (RL-1042, Vector Laboratories)^23^ in the vitelline vein using a glass microcapillary needle before being sacrificed. The injected lectin was allowed to circulate for 10 min before these embryos were sacrificed. For the timeline experiment (Supplementary Fig. 4B), the volume of injected LCA was adjusted based on the age of the embryos: 10 µL was injected in day 5–7 embryos, 12 µL in day 8–9 embryos, 15 µL in day 10 embryos and 20 µL in day 11 embryos. To retrieve the organoids, the abdominal wall of the embryos was opened along the longitudinal axis using forceps under a stereo microscope. Organoids were localized based on tissue morphology and removed using micro scissors and a surgical knife.

### Immunofluorescence analysis

Organoids in vitro were fixed in 2% paraformaldehyde (PFA) at 4 °C for 20 min. Transplanted organoids were fixed using 4% PFA at 4 °C overnight and thoroughly washed with PBS. They were used for whole mount staining. Non-transplanted organoids or transplanted tissues were permeabilized and blocked in 0.3% TritonX in PBS containing 10% donkey serum for 2 h. Primary antibodies were diluted in blocking solution and incubated 24–72 h. Upon washing, secondary antibodies were incubated for 2–4 h at room temperature. Kidney organoids were characterized for NPHS1 (AF4269, dilution 1:100), CD31 (555444, dilution 1:100), ECAD (610181, dilution 1:300), LTL-biotin (B-1325, dilution 1:300), and PDGFRβ (MAB1263, dilution 1:50). Primary antibodies were detected with donkey-α-sheep Alexa Fluor 568 (A-21099, dilution 1:500) and 647 (A-21448, dilution 1:500), donkey-α-mouse Alexa Fluor 405 (ab175658, dilution 1:500) and 488 (A-212-02, dilution 1:500), streptavidin Alexa Fluor 532 (S11224, dilution 1:200) and 647 (S21374, dilution 1:200) (Supplementary Table 1). All antibodies and isotype controls were validated in human kidney samples. Nuclei were stained with Hoechst33258 (Thermo Fisher Scientific) and tissues embedded in ProLong Gold Antifade Mountant (Thermo Fisher Scientific) in 35 mm glass bottom dishes (MatTek corporation) or adhesive microscope slides (StarFrost, Knittel glass). Leica White Light Laser Confocal Microscope TCS SP8 using LAS-X Image software with 3D module (Leica) was used for analysis of the tissues.

### Quantification of CD31 positive endothelial cells

Quantification of CD31 positive endothelial cells was performed in whole mount half or whole untransplanted and transplanted organoids at d7+19–20. Imaris Software was used to calculate the volume of CD31 positive cells as a percentage of whole organoid volume. To select the organoid tissue, a surface was generated manually by drawing a contour around the organoids every 5 slides of the Z-stack. This surface was used to calculate the volume of the organoid with the statistics function in Imaris. Next, a surface was created for the CD31 channel, and the volume of this surface was obtained in the same manner. In case of transplanted organoids with high background signal in the surrounding chicken tissue, a mask was created for the CD31 channel based on the previously generated organoid surface. This masked CD31 channel was then used to create a surface and calculate the CD31 volume. Threshold and minimum number of voxels for CD31 surface generation were set separately for each sample, to be able to correct for differences in background and if necessary, background falsely identified as endothelial cells by the software was removed manually. This analysis was performed for 6 untransplanted organoids, all from different differentiation batches (3 from iPSC-MAFB, 3 from LUMC0072) and 6 matched transplanted organoids. Graphpad Prism 9.0.1 was used for statistical analysis. Means were compared between groups using an unpaired two-tailed t-test. The normality of the distribution was tested and confirmed using the Shapiro–Wilk and Kolmogorov–Smirnov test. Individual data points and mean (SD) are presented.

### Single cell RNA sequencing sample preparation

Untransplanted and transplanted (on day 7+12) organoids from the same differentiation of the hiPSC-MAFB line were dissociated to single cells at day 7+13 and d7+20 using a collagenase I buffer consisting of 600U ml^−1^ collagenase Type I (Worthington) and 0.75 U ml^−1^ DNAse (Sigma Aldrich) in HBSS with calcium and magnesium (Thermo Fisher Scientific) followed by a TrypLE buffer consisting of 5U ml^−1^ DNAse I (Sigma Aldrich) and 4 µg ml^−1^ heparin (Sigma Aldrich) in 80% TrypLE select 10× (Thermo Fisher Scientific) in DPBS (Thermo Fisher Scientific). 5 organoids were placed in Collagenase I buffer and incubated in a water bath at 37 °C for 40 min with repeated pipetting. The cell suspensions were centrifuged at 300G, and the cell pellet was incubated at 37 °C for another 5 min in TrypLE buffer. The dissociation was stopped by adding cold HBSS+/+ with 10% FCS, and the suspension further diluted with HBSS+/+. The single cell suspension was centrifuged at 400 G and the cell pellet was resuspended in PBS+0.1% BSA. All cells from organoids from the same condition were pooled (d7+13 untransplanted: 94 organoids, transplanted: 75 bisected organoids (transplanted in 150 chicken embryos) d7+20 untransplanted: 95 organoids, transplanted: 46,5 bisected organoids (transplanted in 93 chicken embryos)) and 30 mL of PBS + 0.1%BSA was added to enable cell counting. The single cell suspensions were converted to barcoded scRNA-seq libraries with a targeted cell recovery of 9000 cells/condition using the Chromium Single Cell 3’ v3 Library, Gel Bead & Multiplex Kit and Chip Kit (10x Genomics).

### Deep sequencing and data pre-processing

The scRNA-seq libraries were converted into DNA nanoballs (DNB) using a standard circularization protocol optimized for the DNBSEQ-T7 sequencer, and all subsequent steps were carried out following the standard operation procedure^68,69^. The scRNA-seq libraries were sequenced aiming for at least 50,000 reads/cells, and using 28+100+8 bp paired‐end sequencing to determine (1) the cell barcode and UMI, (2) the transcript and (3) the sample index, respectively. Demultiplexing according to the sample barcodes, and subsequent read alignment were performed using Cell Ranger (10x Genomics, v3.1.0). For untransplanted kidney organoid samples, reads were aligned to human genome (GRCh38) only. For transplanted kidney organoid samples, a combined genome including human (GRCh38) and chicken (GRCg6a) genomes, was prepared using the *mkgtf* and *mkref* commands from Cell Ranger (10X genomics), following 10X genomics instructions. Reads associated to these samples were then aligned to this reference. Key sequencing metrics are summarized in Supplementary Data 1.

Raw unfiltered data matrices from the Cell Ranger output were then further processed with R (v4.1.1) and Seurat package (v4.0.4). For multi-species samples, the percentage of genes belonging to each species was determined using the *PercentageFeatureSet* function from Seurat package, and cells were labeled either “human” or “chicken” when >85% of total genes were mapped to the respective species, whereas ambiguous cells (unlabeled) were discarded. Cells from multi-species samples were then segregated per species for downstream processing, resulting in both a human and a chicken dataset, for each transplanted kidney organoid sample. The following quality control steps were performed for each dataset: (i) genes expressed by less than 10 cells were removed; (ii) cells that expressed fewer than 600 genes (for human datasets) and 200 genes (for chicken datasets) were discarded as low-quality cells (Supplementary Data 1); (iii) cells with a detected number of genes exceeding a “doublet” threshold as listed in Supplementary Data 1 were excluded (determined by inspecting the cell frequency per total number of genes expressed, for each sample); (iv) cells with a fraction of mitochondrial genes >15% (for human datasets) and >5% (for chicken datasets) were also removed (cells with compromised cell membrane/dying or dead cells). After pre-processing, human cells-containing samples were merged as well as chicken cells-containing samples, resulting in 24,988 human cells (27,349 human cells before removal of dying/dead cells), and in 8018 chicken cells (8279 chicken cells before removal of dying/dead cells).

### ScRNAseq data analysis

Data were normalized using the *NormalizeData* function from Seurat Package, and the top 2000 highly variable genes were identified using *FindvariableFeatures*. Data were scaled with the *Scaledata* function and reduced with principal component (PC) analysis using *RunPCA* function. The top 30 PCs (40 PCs for chicken cells) were used for visualization using uniform manifold approximation and projection (UMAP) with the *RunUMAP* function as implemented in Seurat. Next, a shared nearest-neighbor graph (SNN) was determined using *FindNeighbors* function, and used to calculate clusters with the *FindClusters* function. Top marker genes were calculated with the *Findallmarkers* function using default settings. Remaining low-quality cell and doublet clusters were characterized by a low number of genes expressed per cell, and an overlap of different cell type markers with high number of genes expressed per cell, respectively, and removed from the dataset. High-quality cells were split according to condition (transplantation & timepoint) and aligned using *Seurat* integration tool: *FindintegrationAnchors* and *IntegrateData* functions, with 2000 anchors and 40 PCs as settings. Clustering was performed as described above using 40 PCs and main cell types for human and chicken datasets were identified. For the human dataset, nephron cell (*NPHS1, NPHS2, MFAB, WT1, EPCAM, KRT19, …*) and mesenchymal cell clusters (*COL1A1, COL1A2, COL3A1, TAGLN, PDGFRA*, …) were subsetted using the *subset* function for more in-depth analysis. Briefly, for both the mesenchymal and nephron cell clusters, 2000 highly variable genes were identified, and data were integrated per condition (transplantation & timepoint) using 2,000 anchors and 40 PCs as settings, as described above. Integrated data were further scaled, reduced within 40 PCs that were further used for UMAP visualization, and clustering, as described above. Upregulated markers were identified using the *FindAllMarkers* function, including genes for which Log2FC > 0 only, and using other parameters as default. Cluster identity was determined according to cell-(sub)type specific top marker genes: podocyte markers as *MAFB, NPHS1, NPHS2, WT1*, epithelial cell markers a*s EPCAM* and *KRT19* for tubular epithelial cells, and fibroblast markers as *COL1A1, COL1A2, COL3A1*, smooth muscle cell markers as *ACTA2*, *TAGLN*, *MYL9*, pericyte/mesangial cell markers as *REN, GATA3, PDGFRB*, mesenchymal progenitor markers as *PRRX1* and *PRRX2*, neural-like cell markers as *NTREK2, METRN, GAP43, STMN2*, chondrocyte-like cell markers as *COL9A1, COL9A2, COL9A3, MATN4*, and melanocyte-like cell markers as *PMEL, MITF, MLANA*. As for the chicken cells, the following markers were used to identify clusters: erythroid cells (*HBA1, HBAD,* and *HBE1*), megakaryocytes (*GP9, ITGB3,* and *GP1BB*), endothelial cells (*PECAM1, CDH5,* and *PROX1*), macrophages (*CD74* and *C1QB*), granulocytes (*DEFB4A and AvBD1*), lymphoid cells (*CD3D* and *IGLL1*), fibroblasts (*COL3A1* and *COL14A1*), pericytes (*KCNJ8, RGS5,* and *ABCC9*), smooth muscle cells (*ACTA2, TAGLN,* and *ACTG2*), hepatic stellate cells (*COLEC11* and *HGF*), epithelioid cells (*KRT24* and *KRT1*), hepatocytes (*ALB, FGB,* and *FGG*), Leydig cells (*CYP11A1, STAR,* and *HSD3B1*), Sertoli cells (*AMH* and *SOX9*), Schwann cells (*NRN1, SOX10,* and *CRYAB*) and neurons (*GAP43, NEFM,* and *NSG1*). Proliferative cell clusters were identified as either within G1/S phases or G2/M phases according to canonical marker genes, as listed in Seurat.

#### Dot plot and heatmap visualization

All dotplots were prepared using *DotPlot* function as implemented in Seurat. All heatmaps were prepared with BIOMEX software (v1.5 - https://carmelietlab.sites.vib.be/en/biomex)^70^, except heatmaps displaying gene expression of ligand and receptors, which were prepared with the ComplexHeatmap package (v2.11.1) in R. For heatmaps prepared in BIOMEX, briefly, count data matrix was exported from the Seurat object using the *GetAssayData* function (selected assay “RNA”) and uploaded in BIOMEX altogether with the corresponding metadata information. Data were normalized (“Standard” parameter) and (auto)scaled for heatmap visualization within BIOMEX. Cluster-averaged gene expression was used to account for cell-to-cell transcriptomic stochastics. Top marker gene heatmaps were prepared, including uniquely upregulated genes resulting from the marker gene identification tool from BIOMEX. For heatmaps prepared with the ComplexHeatmap package, briefly, the averaged expression profiles per each cluster and for all genes were calculated using the *AverageExpression()* function from Seurat package. Next, scaled data were obtained from the RNA assay using *GetAssayFunction()* function and selected gene data were extracted from the resulting matrix. These values were used as input for the *Heatmap()* function from the ComplexHeatmap package.

#### Cluster similarity analysis

Hierarchical clustering was performed with BIOMEX using highly variable genes as identified in BIOMEX, with Euclidean distance and complete linkage. The confidence of each branch of the tree was estimated by the bootstrap resampling approach from the R-package *pvclust*^71^, using a confidence score of >0.05. Number of bootstrapping was set at 1000.

#### Metadata quantification

Metadata from the Seurat object were exported, as described above and uploaded in BIOMEX. Metadata were quantified and visualized using the metadata quantification tool, as implemented in BIOMEX. “barplots” was used as plot setting.

#### Differential gene expression analysis (DGEA)

For DGEA according to the transplantation condition, the *FindMarkers* function from Seurat was used on the “RNA” assay. Differential expression was run with the MAST package. The number of UMIs per cell was used as latent variable to correct for unequal sequencing depth between cells.

#### Geneset enrichment analysis (GSEA)

DGEA outputs were used for GSEA using the *ClusterProfiler* package (v4.0.5). GSEA was performed on gene ontology genesets (biological processes only) with *gseGO* function, as implemented in *ClusterProfiler*. Genesets including less than 10 genes were not included. All other parameters were used as default. In order to remove as much as possible redundant terms, results were simplified using the online Revigo tool (http://revigo.irb.hr) for both significantly up- and downregulated genesets with Normalized Enrichment Scores used as ranking method. The resulting list was aimed to be small (selected 0.5 parameter). Output from Revigo was exported and plotted using *ggplot* function, as implemented within the *ggplot2* package (v3.3.5).

#### Ligand/Receptor interaction analysis (L/R analysis)

For L/R analysis, *CellChat* package (v1.1.3) was used. First, the melanocyte-like cluster was removed from the analysis since it was absent from the transplanted d7+20 kidney organoid condition. The normalized data matrix “data” slot, from the “RNA” assay within the Seurat object was extracted for each condition (d7+20 condition, and the other conditions as pooled, or taken separately for the comparison of the total number of interactions and interaction probabilities between the four conditions) using *GetAssayData()* function from the Seurat package. The metadata containing final cluster identities were extracted as a data frame. Data and metadata were used to create CellChat objects using *createCellChat()* function. The complete human cellchat database was used. Expression data was subsetted to genes present in the database using *subsetData()* function and overexpressed genes and interactions were identified for each condition with *identifyOverExpressedGenes()* and *identifyOverExpressedInteractions()* functions, respectively. Communication probabilities and inference of the cellular communication network were calculated using *computeCommunProb()*, *computeCommunProbPathway()*, *aggregateNet()* and *netAnalysis_computeCentrality()* functions. Cellchat objects were merged with the dedicated *mergeCellChat()* function. Comparison of the total number of interaction and interaction strength was performed using *compareInteraction()* function setting the “measure” argument as “weight” when appropriate. The *netVisual_heatmap()* function, setting the “measure” argument as “weight”, was used to generate a heatmap of differential interaction strength between cell populations. To identify the upregulated signaling ligand-receptor pairs between ECs and pericytes/mesangial cells, we compared the communication probabilities between conditions and visualized the results as a dot plot using the *netVisual_bubble()* function, with “max.dataset” argument set as “2”.

### Transmission electron microscopy (TEM)

For morphological analysis, small sections of in vitro kidney organoids and transplanted organoids were fixed for 2 h at room temperature in 1.5% glutaraldehyde (Electron Microscopy Sciences) in 0.1 M sodium cacodylate buffered solution (pH 7.4). The tissues were further rinsed with sodium cacodylate buffer and fixed in a solution of 1% osmium tetroxide (Electron Microscopy Sciences) in 0.1 M sodium cacodylate buffer for 1 h on ice. Afterwards, samples were washed with sodium cacodylate buffer and dehydrated in a series of 70, 80, 90, and 100% ethanol. The probes were infiltrated with a mixture of 1:1 Epon LX-112 (Ladd Research) and propylene oxide (Electron Microscopy Sciences) for 1 h, followed by infiltration with pure Epon for 2 h. Subsequently, the samples were embedded in pure Epon, mounted in BEEM capsules (Agar Scientific) and polymerized for 48 h at 60 °C. Ultrathin sections (100 nm) were collected onto copper slot grids (Storck Veco B.V.), covered with formvar film and a 7 nm carbon layer. The sections were contrasted with an aqueous solution of 7% uranyl acetate for 20 min, followed by Reynolds lead citrate for 10 min. The imaging was performed at an acceleration voltage of 120 kV with a FEI Tecnai G^2^ Spirit BioTWIN TEM (FEI), equipped with an Eagle 4 K slow-scan charge-coupled device (CCD) camera (FEI). Data were collected at 18,500× magnification, corresponding to a 1.2 nm pixel size at the specimen level. Large virtual slides showing glomerular structures were acquired using automated large-scale data collection combined with stitching software^18^. These extensive digital images provide an overview of entire glomeruli and the possibility to zoom in to high detail, allowing for qualitative analysis.

### Serial Block Face Scanning Electron Microscopy (SBF-SEM)

For SBF-SEM analysis, 2 transplanted organoids and 1 untransplanted organoid, all generated using iPSC-MAFB were used. Kidney organoid fixation, staining and embedding was done according to an adapted protocol from Deerink et al.^72^ Samples were fixed for 2 h on ice with 2.5% glutaraldehyde and 2% paraformaldehyde (Electron Microscopy Sciences) in 0.15 M sodium cacodylate buffer containing 2 mM calcium chloride. A first post-fixation step was performed for 1 h on ice, using a solution of 2% osmium tetroxide and 1.5% potassium ferrocyanide in 0.15 M sodium cacodylate buffer containing 2 mM calcium chloride. Subsequently samples were treated for 20 min at room temperature with 1% aqueous solution of thiocarbohydrazide, followed by 2% aqueous osmium tetroxide for 30 min at room temperature and incubated overnight at 4 °C in a 1% aqueous solution of uranyl acetate. The probes were then stained for 30 min at 60 °C, using Walton’s lead aspartate, and dehydrated in series of 20% up to 100% ethanol, followed by dehydration in anhydrous acetone. Tissue samples were infiltrated in mixtures of 1:3, 1:1, 3:1 Durcupan ACM resin (Ladd Research) and acetone, in 2 h steps, then overnight at room temperature in 100% Durcupan. A final infiltration step with fresh Durcupan was performed for 2 h at room temperature. The samples were then placed in BEEM capsules, embedded in Durcupan and polymerized for 48 h at 60 °C. Sample blocks were trimmed and 300 nm thick sections were cut and stained with toluidine blue for light microscopy to find regions of interest containing glomerular structures. For SBF-SEM, small blocks of approximately 800 × 800 × 400 µm were trimmed and glued on aluminum pins (Gatan) using Conductive Silver Epoxy Kit (Agar Scientific), then coated with a thin layer of gold right before imaging. A data set from transplanted and non-transplanted organoid was collected on a GeminiSEM 300 microscope (ZEISS) equipped with a 3View 2XP system (Gatan). The SEM was operated at a chamber pressure of 5 Pa, in variable pressure mode, using an acceleration voltage of 1.6 kV and the 60 µm aperture. Back-scattered electron images were acquired using the OnPoint BSE detector (Gatan). 4 datasets were generated of untransplanted glomerular structures and 3 of transplanted glomerular structures, of which 2 (1 untransplanted and 1 transplanted) were used for 3D visualization. The data set from the non-transplanted organoid was acquired at 10,000× magnification, with a pixel size of 9 nm, a dwell time of 2.8 µs and a 50 nm slice thickness. The sample size was 61 × 61 × 28 µm, and the image size 6800 × 6800 pixels × 557 slices. The data set from the transplanted kidney organoid was acquired at 9000× magnification, a pixel size of 10 nm, a dwell time of 1.5 µs and a 60 nm slice thickness. The sample size was 65 × 75 × 27 µm, and the image size 6500 × 7500 pixels × 451 slices. SBF-SEM image stacks were acquired as dm4 files and aligned using Digital Micrograph software (Gatan).

### Annotation and visualization of SBF-SEM data

2 SBF-SEM datasets (1 untransplanted and 1 transplanted) were annotated using artificial intelligence-based image segmentation by Ariadne (Ariadne-Service GmbH). In short, nuclei, extracellular space (ECS), and plasma membrane labels were predicted using a convolutional neural network (CNN) architecture based on the U-Net^73^. Stochastic weight averaging was used in the ER prediction^74^. Somata were segmented by running the watershed algorithm on the plasma membrane probability map, using the predicted nucleus locations as seeds and excluding the ECS regions. CNNs were implemented in the ELEKTRONN deep learning library (www.elektronn.org). Image stacks of segmented structures (~12 Gb or ~25 Gb in size) were binned (in X and Y) using Fiji^75^ or IMOD^76^ to decrease the file size, and converted to mrc stacks and the headers were corrected using IMOD. Cell types were annotated by hand according to morphology and location and surfaces were smoothed, simplified and visualized using AMIRA 3D software for life sciences (Thermo-Fisher Scientific). Surfaces were imported into Cinema4D (Maxon) for final rendering using Redshift (Maxon).

### Reporting summary

Further information on research design is available in the Nature Research Reporting Summary linked to this article.

## Supplementary information

Supplementary information

Supplementary movie 1

Supplementary movie 2

Supplementary movie 3

supplementary movie 4

Supplementary movie 5

Supplementary movie 6

Supplementary data 1

Supplementary data 2

Supplementary data 3

Supplementary data 4

Supplementary data 5

Supplementary data 6

Supplementary data 7

Supplementary data 8

Supplementary data 9

Supplementary data 10

Supplementary data 11

Related Manuscript File

REPORTING SUMMARY

## Data Availability

The source data underlying Figs. 1f, h, 2d, h, 3a and Supplementary Fig. 6a, b are provided as a source data file. Both raw and processed sequencing data are available in ArrayExpress under accession number E-MTAB-11429. Other data are available from the authors upon request.

## Code availability

Custom code is available upon reasonable request.
