# Supplementary material for: Vasculogenesis in kidney organoids upon transplantation
Source: NPJ Regen Med. 2022 Aug 19;7:40. doi: 10.1038/s41536-022-00237-4 (PMC9391397; doi:10.1038/s41536-022-00237-4)
Supplement: Supplementary file 1 — Supplementary information [file 41536_2022_237_MOESM1_ESM.pdf]

Supplementary figure 1

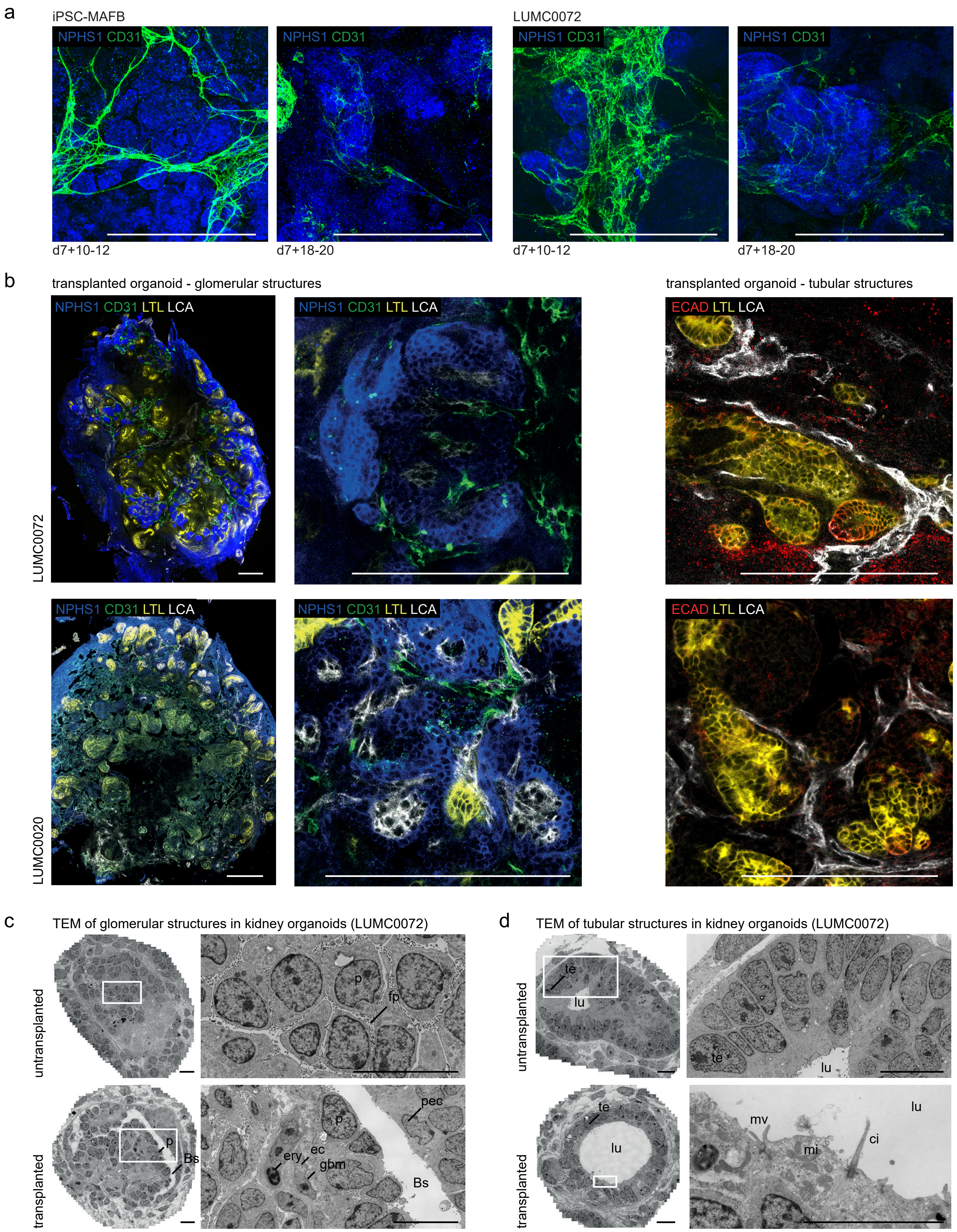

Supplementary figure 1 – Vascularization of untransplanted and transplanted kidney organoids in multiple cell lines

a. Maximum projection images of Z-stacks of untransplanted kidney organoids derived from hiPSC-MAFB and LUMC0072 at d7+10-12 and d7+18-20 of differentiation. Endothelial cells are more abundant at the earlier time point. Scalebar 200μm.
b. Kidney organoids derived from LUMC0072 and LUMC0020 are vascularized upon transplantation for 8 days. Glomerular structures (NPHS1+, blue) are invaded by perfused capillaries (LCA+, white and CD31+, green) and proximal (LTL+, yellow) and distal (ECAD+, red) tubular structures are aligned by them. Scalebar 200μm.
c. TEM imaging of kidney organoids derived from LUMC0072 demonstrates increased maturation of glomerular structures upon transplantation for 8 days. Podocyte clusters are invaded with capillaries containing erythrocytes, parietal epithelial cells form a Bowman's capsule, a glomerular basement membrane is deposited between the podocytes and endothelial cells. *Bs* Bowman's space*, ec* endothelial cell*, ery* erythrocyte, *fp* foot process*, gbm* glomerular basement membrane*, p* podocyte*, pec* parietal epithelial cell. Scalebar 10μm.
d. TEM imaging of kidney organoids derived from LUMC0072 demonstrates increased maturation of tubular structures. Tubular epithelial cells have formed a monolayer and their nucleus has moved toward the basolateral side of the cell. A wide tubular lumen is visible. Cilia, microvilli and abundant mitochondria are visible. *ci* cilium, *lu* lumen, *mi* mitochondrion, *mv* microvilli, *te* tubular epithelium. Scalebar untransplanted overview and magnification and transplanted overview 10μm. Scalebar transplanted magnification 5μm.

Supplementary figure 2

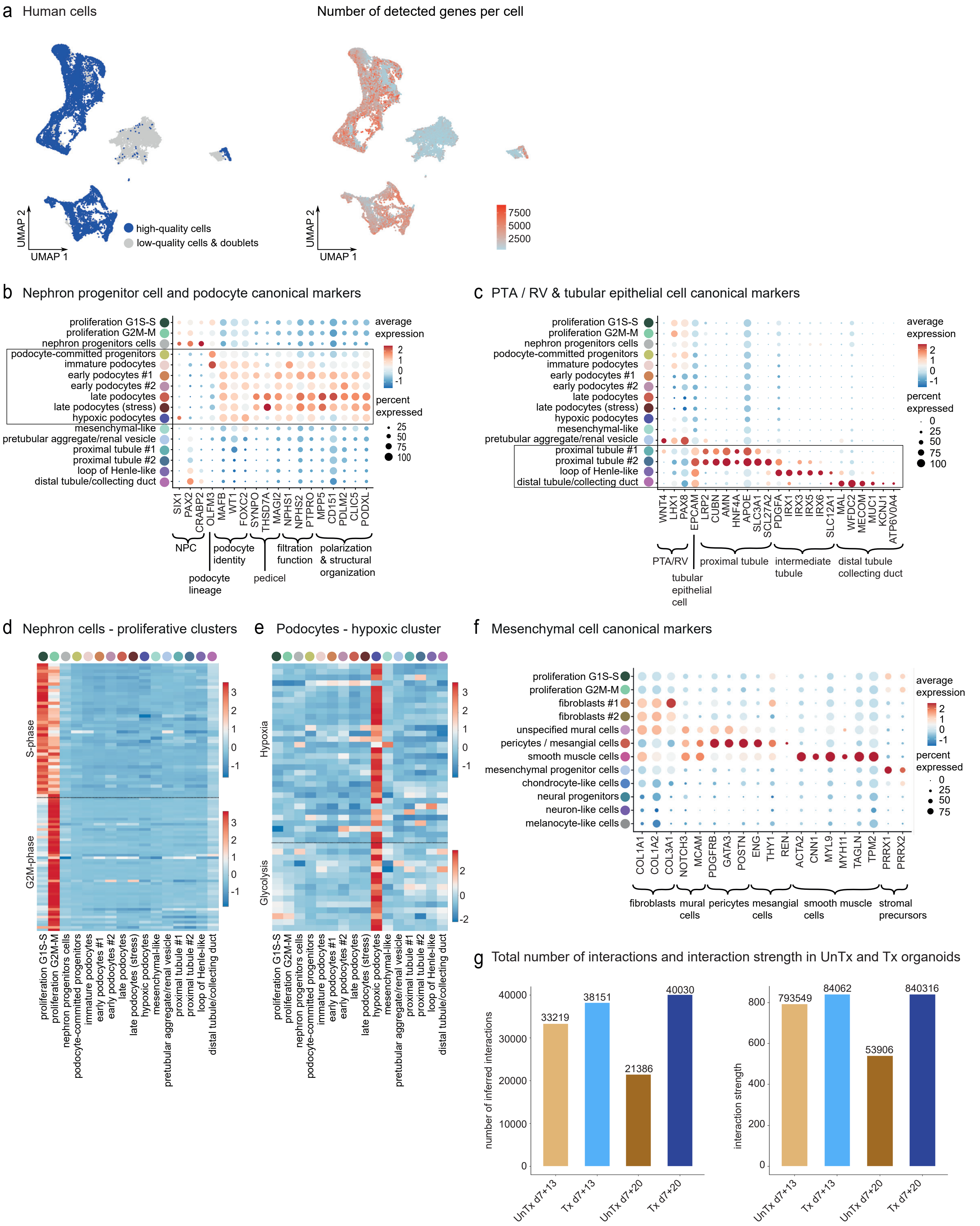

Supplementary figure 2 – Selection of high quality organoid cells and subclustering of nephron and mesenchymal cell types

a. UMAP visualization of (left) all human cells and selection of high-quality cells (high-quality cells: blue, low-quality cells and doublets: grey), and (right) number of detected genes per cell.
b. Dot plot of canonical nephron progenitor cell (NPC) and podocyte markers used to characterize podocyte cluster identity. Dot size indicates proportion of cells in cluster expressing a gene, colour intensity indicates the level of expression
c. Dot plot of canonical renal vesicle / pretubular aggregate (RV/PTA) and tubular epithelial cell markers used to characterize tubular epithelium cluster identity. Dot size indicates proportion of cells in cluster expressing a gene, colour intensity indicates the level of expression
d. Expression-level scaled heatmap of S-phase and G2M-phase genes, upregulated in nephron proliferation clusters. Scale: z-score of the gene expression level.
e. Expression-level scaled heatmap of hypoxia and glycolysis-related genes, upregulated in the hypoxic podocytes clusters. Scale: z-score of the gene expression level.
f. Dot plot of canonical mesenchymal cell markers used to characterize cluster identity. Dot size indicates proportion of cells in cluster expressing a gene, colour intensity indicates the level of expression
g. Bar plot of the total number of inferred interactions (left) and total interaction strength (right) in transplanted and untransplanted organoids at different timepoints (d7+13 and d7+20).

Supplementary figure 3

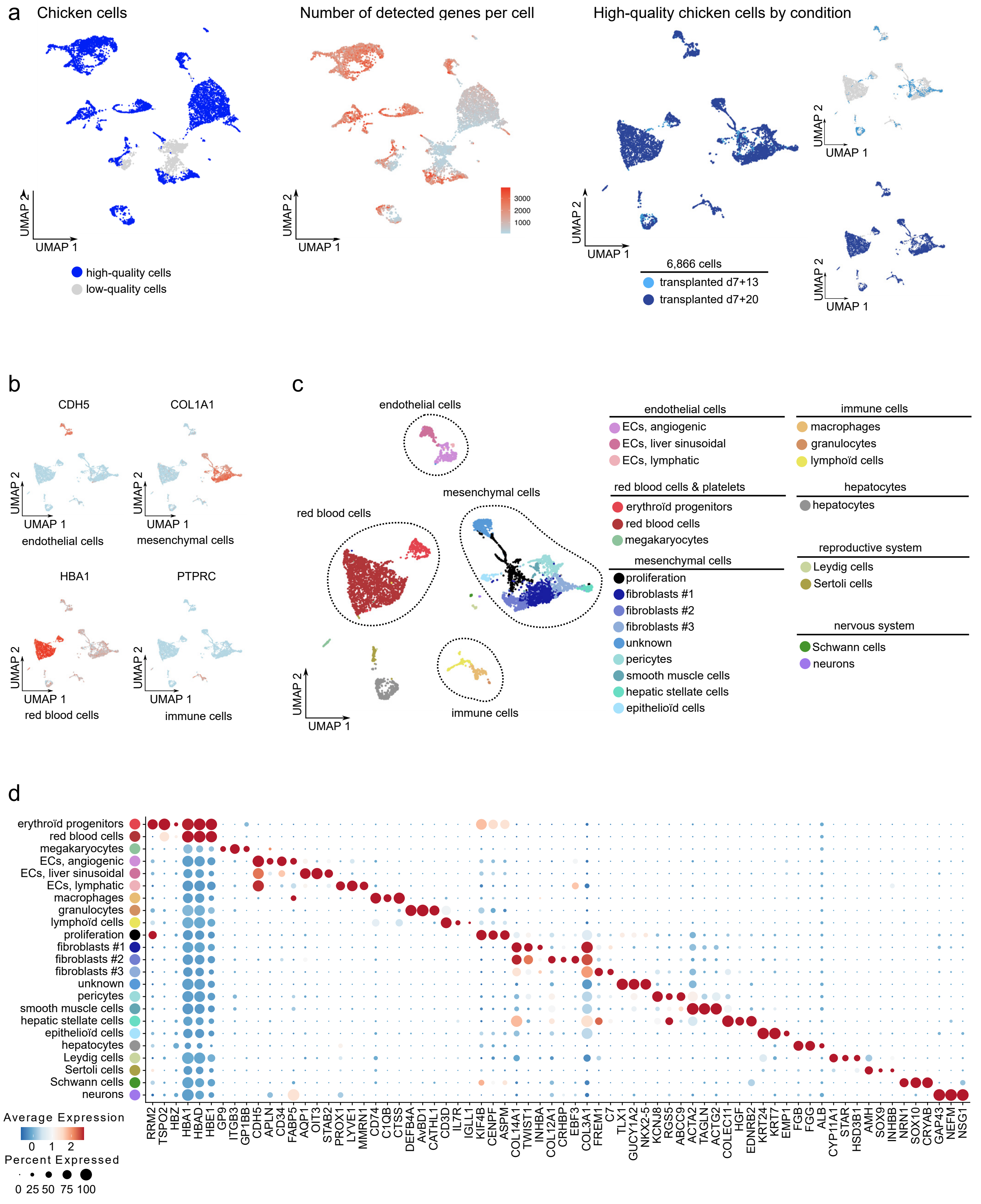

Supplementary figure 3 –Selection and subclustering of high-quality chicken cells

a. UMAP visualization of all chicken cells color-coded by (left) cell quality (high= blue, low= grey), (middle) number of detected genes per cell. (Right) UMAP visualization of a total of 6,866 high-quality chicken cells from transplanted kidney organoids color-coded by timepoint condition (857 cells from d7+13, 6,009 cells from d7+20). Note that these cells are partly derived from the organoid itself and partly from chicken tissue attached to the organoid.
b. UMAP visualization of all high-quality chicken cells, color-coded by the expression level of *CDH5*, *COL1A1*, *HBA1*, and *PTPRC* genes, canonical markers for endothelial cells, mesenchymal cells, red blood cells and immune cells (red: high expression level, blue: low expression level).
c. UMAP visualization of all high-quality chicken cells, color-coded by identified chicken cell clusters: endothelial cells (n=3 clusters), red blood cells & platelets (n=3 clusters), mesenchymal cells (n=9 clusters), immune cells (n=3 clusters), hepatocyte (n=1 cluster), cells from the reproductive system (n=2 clusters) and cells from the nervous system (n=2 clusters).
d. Dot plot representing canonical marker gene expression in chicken cell clusters. Dot size indicates proportion of cells in cluster expressing a gene, colour intensity indicates the level of expression.

Supplementary figure 4

a

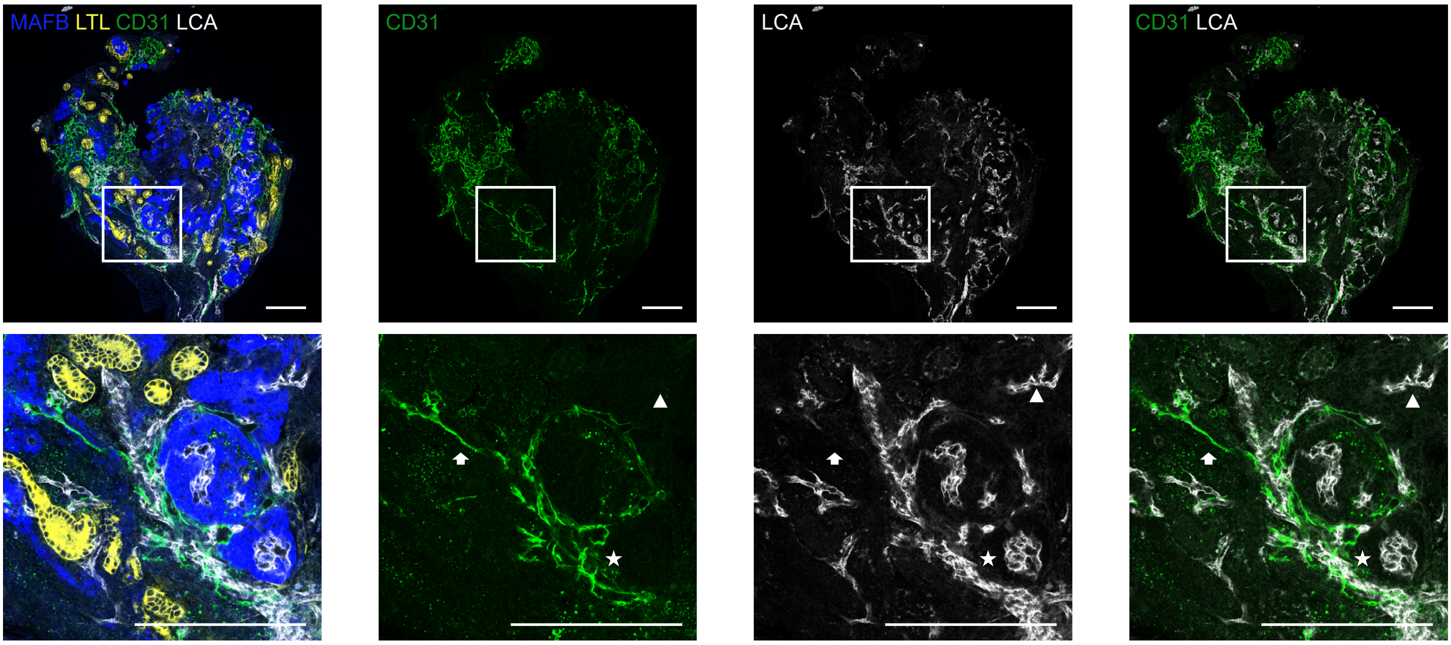

b Timeline of vascularization after transplantation

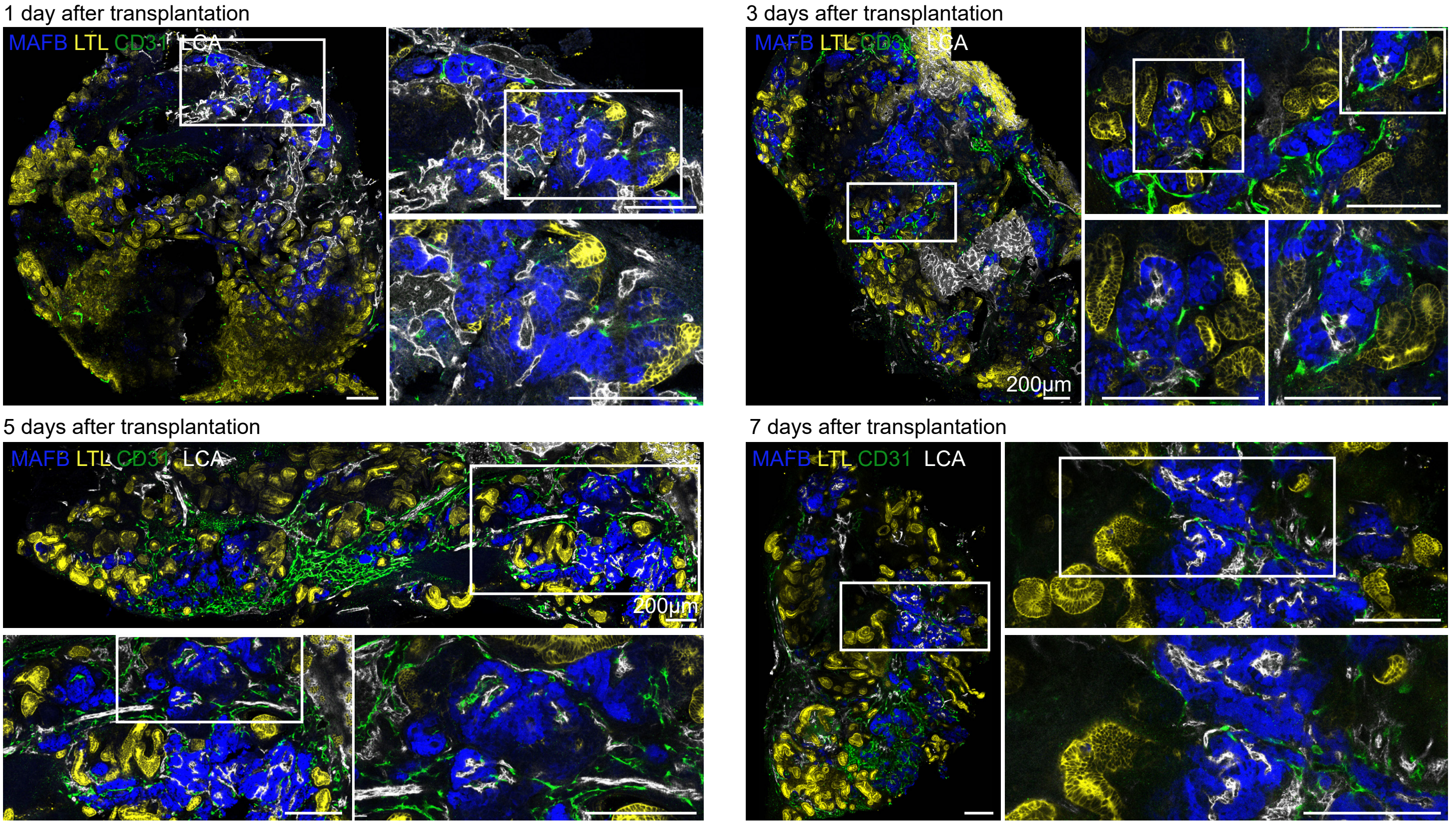

Supplementary figure 4 – Origin of endothelial cells and timeline of vascularization

a. Combined staining for human CD31 (green) and injected LCA (white) demonstrates the presence of an extensive perfused vascular network in transplanted organoids d7+19-20. Three types of blood vessels can be distinguished: perfused human organoid derived endothelial cells, (CD31+, LCA+) unperfused human organoid derived endothelial cells (CD31+, LCA-), and perfused chicken derived endothelial cells (CD31-, LCA+). Examples are marked in the magnifications of the boxed areas by a star (perfused human), arrow (unperfused human), and arrowhead (perfused chicken). Scalebar 200µm.
b. Timeline demonstrating the rapid vascularization of kidney organoids upon transplantation. On day 1 after transplantation, blood vessels have entered the organoid, but not the glomerular structures. On day 3, some glomerular structures have become vascularized, which increases markedly on day 5 and 7 after transplantation. Magnifications of the boxed areas are displayed. Scalebar 200µm.

Supplementary figure 5

a Expression of Angiopoietin ligands and receptors by organoid cell clusters

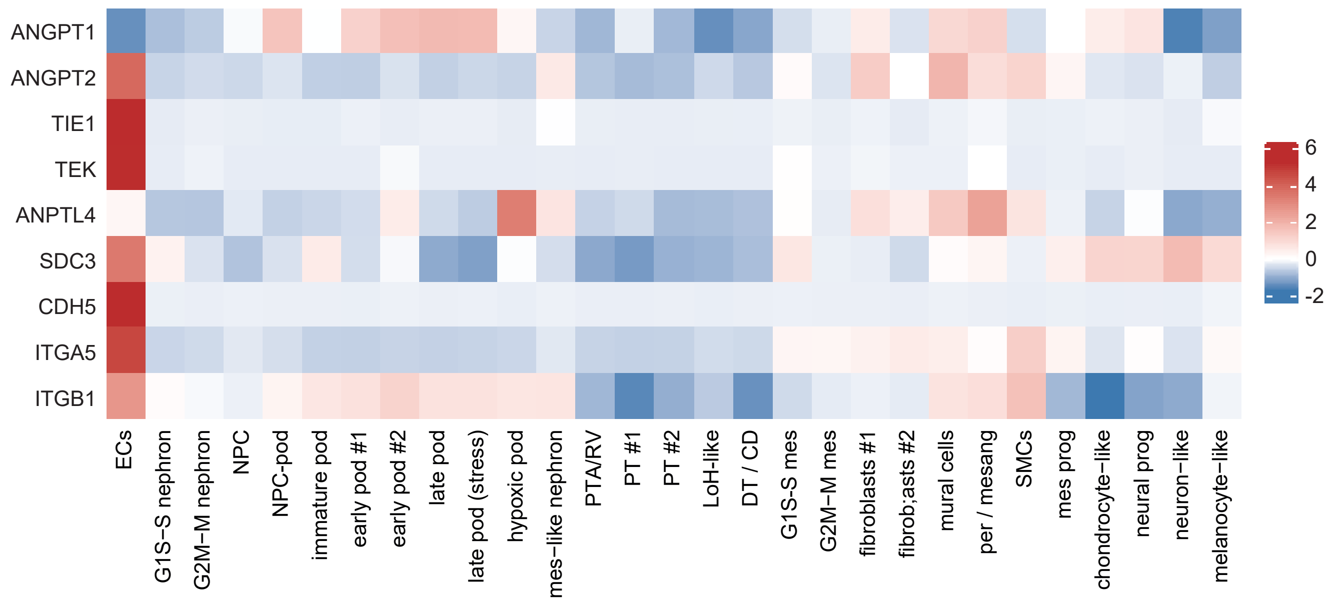

b Expression of PDGF ligands and receptors by organoid cell clusters

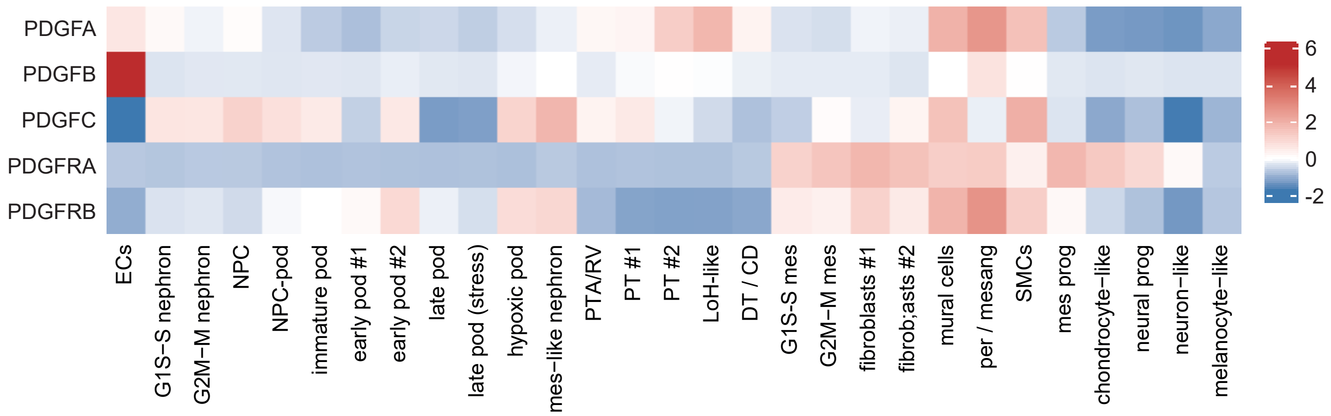

c Expression of VEGF ligands and receptors by organoid cell clusters

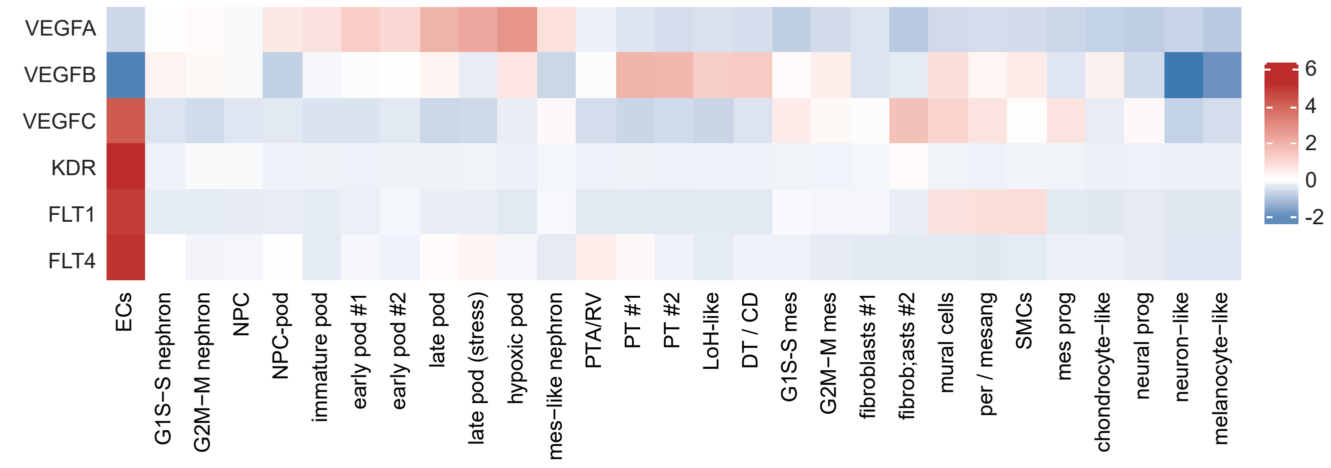

Supplementary figure 5 – Gene expression of ANGPT, PDGF and VEGF ligands and receptors

a. Expression-level scaled heatmap of genes encoding angiopoietin ligands and receptors in organoid cell clusters (cells from all conditions pooled). Scale: z-score of the gene expression level.
b. Expression-level scaled heatmap of genes encoding PDGF ligands and receptors in organoid cell clusters (cells from all conditions pooled). Scale: z-score of the gene expression level.
c. Expression-level scaled heatmap of genes encoding VEGF ligands and receptors in organoid cell clusters (cells from all conditions pooled). Scale: z-score of the gene expression level.

Supplementary figure 6

a GSEA Late podocyte cluster transplanted d7+20 vs untransplanted d7+20

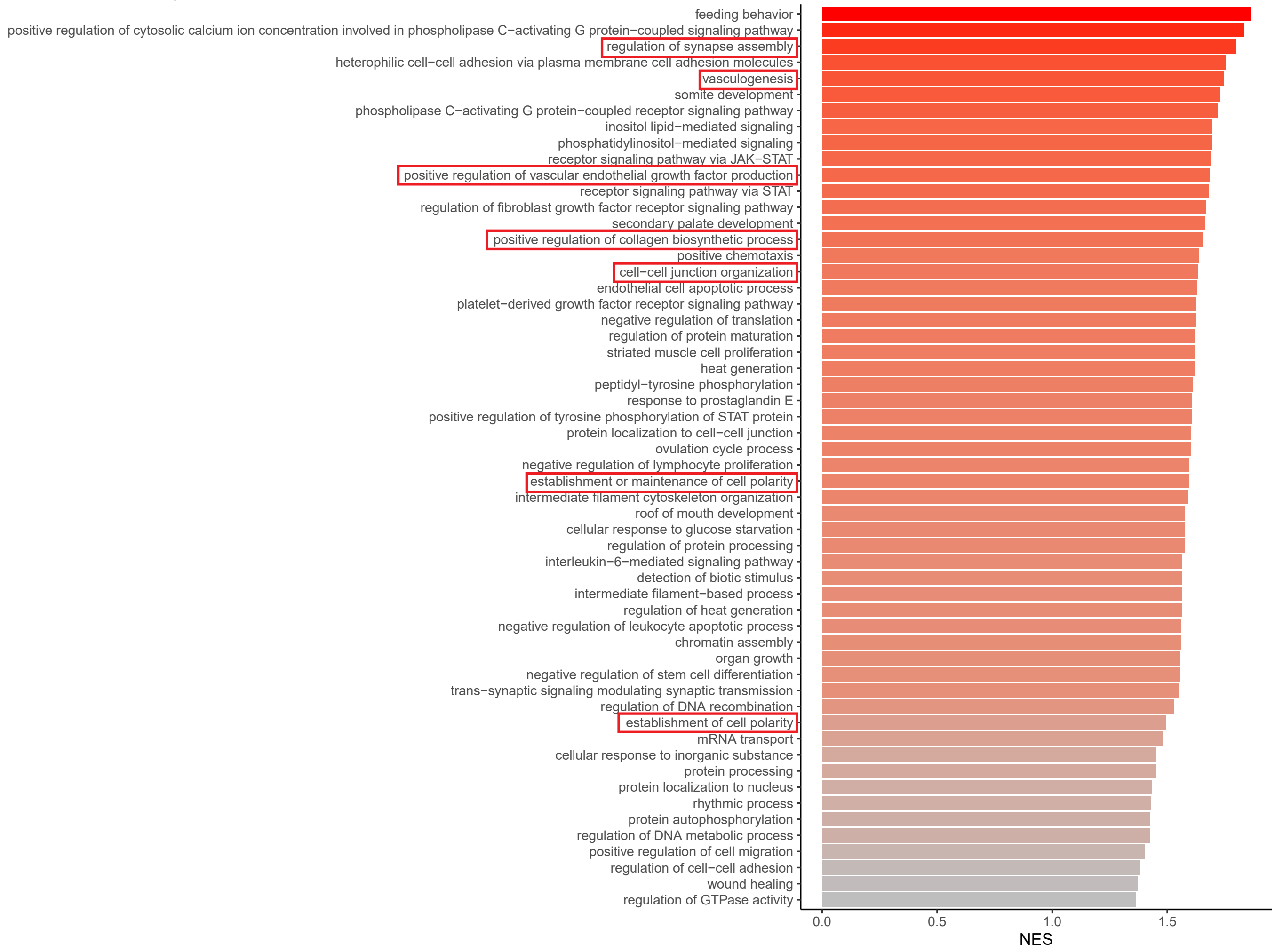

b GSEA All tubular epithelial cells transplanted d7+20 vs untransplanted d7+20

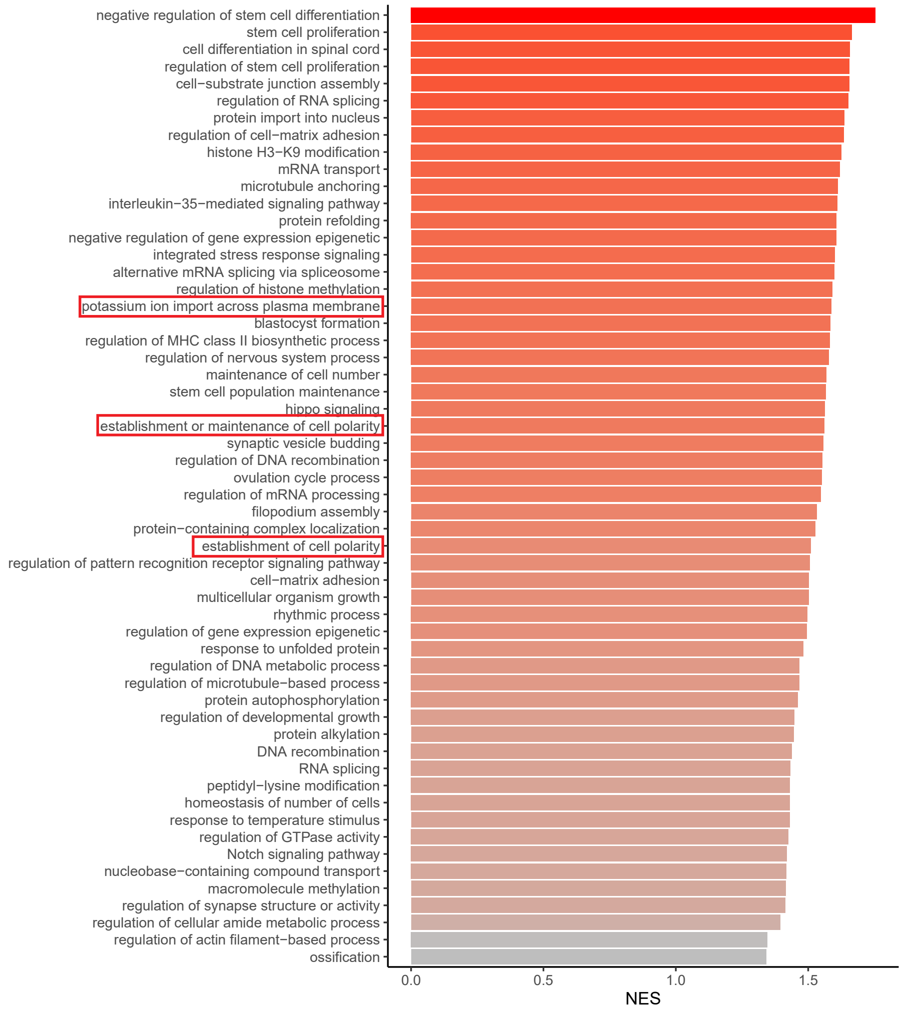

Supplementary figure 6 – Gene set enrichment analysis podocytes and tubular epithelial cells

a. Gene set enrichment analysis (GSEA) for late podocyte cluster in transplanted d7+20 versus untransplanted d7+20 organoids. NES: normalized enrichment score. Source data are provided as a Source Data file.
b. Gene set enrichment analysis (GSEA) for all tubular epithelial cells in transplanted d7+20 versus untransplanted d7+20 organoids. NES: normalized enrichment score. Source data are provided as a Source Data file.

Supplementary table

Supplementary table 1 - Antibodies used for immunofluorescence analysis

Antibody	Type	Supplier	Catalog number	Dilution
Primary antibodies and lectins				
Nephrin - Sheep anti-Human (NPHS1)	Polyclonal	R&D Systems	AF4269	1:100
CD31 - Mouse anti-Human	Monoclonal	BD biosciences	555444	1:100
E-cadherin - Mouse anti-Human (ECAD)	Monoclonal	BD biosciences	610181	1:300
Lotus Tetragonolobus Lectin (LTL)	-	Vector Laboratories	B-1325	1:300
Lens Culinaris Agglutinin rhodamine (LCA)	-	Vector Laboratories	RL-1042-5	1:1
Platelet-Derived Growth Factor Receptor β Mouse anti-Human (PDGFRβ)	Monoclonal	R&D Systems	MAB1263	1:50
Secondary Antibodies				
Alexa Fluor 568 - Donkey anti-Sheep IgG	Polyclonal	Thermo Fisher Scientific	A-21099	1:500
Alexa Fluor 647 - Donkey anti-Sheep IgG	Polyclonal	Thermo Fisher Scientific	A-21448	1:500
Alexa Fluor 405 - Donkey anti-Mouse IgG	Polyclonal	Abcam	ab175658	1:500
Alexa Fluor 488 - Donkey anti-Mouse IgG	Polyclonal	Thermo Fisher Scientific	A-21202	1:500
Streptavidin Alexa Fluor 532 conjugate	-	Thermo Fisher Scientific	S11224	1:200
Streptavidin Alexa Fluor 647 conjugate	-	Thermo Fisher Scientific	S21374	1:200

Supplementary movies

Supplementary movie 1 – Z-stack of SBF-SEM dataset of a glomerular structure inside an untransplanted kidney organoid generated from iPSC-MAFB

The full Z-stack of the SBF-SEM dataset that was used for the 3D reconstruction of the untransplanted glomerular structure demonstrated in Fig. 5B,C and Supplementary Movie 5 is shown. A cluster of podocytes surrounding a central cavity and encapsulated by a layer of parietal epithelial-like cells is visible.

Supplementary movie 2 – Z-stack of SBF-SEM dataset of a glomerular structure inside a transplanted kidney organoid generated from iPSC-MAFB

The full Z-stack of the SBF-SEM dataset that was used for the 3D reconstruction of the transplanted glomerular structure demonstrated in Fig. 5B,C and Supplementary Movie 6 is shown. A perfused capillary containing erythrocytes as well as leukocytes is visible running along the side of the glomerular structure and invading it. The podocytes have reorganized around the capillary and a Bowman's space is visible between the podocyte and parietal epithelial-like cell layer.

Supplementary movie 3 – Z-stack of a second SBF-SEM dataset of a glomerular structure inside an untransplanted kidney organoid generated from iPSC-MAFB

The full Z-stack of a second SBF-SEM dataset of an untransplanted glomerular structure is shown. Again, a cluster of podocytes encapsulated by a layer of parietal epithelial cell like cells is visible. In this glomerular structure, 2 cavities are present that connect to each other. Several other glomerular structures surround the one in the middle. The data set was acquired with SBF-SEM as described in the Materials and Methods section, with a pixel size of 15 nm, a dwell time of 3 µs and a 50 nm slice

thickness. The sample size was 105 x 105 x 25 µm, and the image size 7000 x 7000 pixels x 500 slices.

Supplementary movie 4 – Z-stack of a second SBF-SEM dataset of a glomerular structure inside a transplanted kidney organoid generated from iPSC-MAFB
The full Z-stack of a second SBF-SEM dataset of a transplanted glomerular structure is shown. Again, a perfused capillary is visible invading the glomerular structure, the podocytes have reorganized around the capillary and a Bowman's space is visible between the podocyte and parietal epithelial-like cell layer. The data set was acquired with a pixel size of 12 nm, a dwell time of 3,5 µs and a 60 nm slice thickness. The sample size was 72 x 72 x 18.06 µm, and the image size 6000 x 6000 pixels x 301 slices.

Supplementary movie 5 – 3D reconstruction of SBF-SEM dataset of a glomerular structure inside an untransplanted organoid generated from iPSC MAFB
360° view of the 3D reconstruction shown in Fig. 5 B,C of the SBF-SEM dataset of an untransplanted glomerular structure (Supplementary Movie 1).

Supplementary movie 6 – 3D reconstruction of SBF-SEM dataset of a glomerular structure inside a transplanted organoid generated from iPSC-MAFB
360° view of the 3D reconstruction shown in Fig. 5 B,C of the SBF-SEM dataset of a transplanted glomerular structure (Supplementary Movie 2).
