# Supplementary material for: Vasculogenesis in kidney organoids upon transplantation
Source: NPJ Regen Med. 2022 Aug 19;7:40. doi: 10.1038/s41536-022-00237-4 (PMC9391397; doi:10.1038/s41536-022-00237-4)
Supplement: Supplementary file 20 — REPORTING SUMMARY [file 41536_2022_237_MOESM20_ESM.pdf]

nature portfolio

Corresponding author(s): Marije Koning

Last updated by author(s): Jun 23, 2022

Reporting Summary

Nature Portfolio wishes to improve the reproducibility of the work that we publish. This form provides structure for consistency and transparency in reporting. For further information on Nature Portfolio policies, see our Editorial Policies and the Editorial Policy Checklist.

Statistics

For all statistical analyses, confirm that the following items are present in the figure legend, table legend, main text, or Methods section.

n/a	Confirmed	
☐	☒	The exact sample size (*n*) for each experimental group/condition, given as a discrete number and unit of measurement
☐	☒	A statement on whether measurements were taken from distinct samples or whether the same sample was measured repeatedly
☐	☒	The statistical test(s) used AND whether they are one- or two-sided<br>*Only common tests should be described solely by name; describe more complex techniques in the Methods section.*
☐	☒	A description of all covariates tested
☐	☒	A description of any assumptions or corrections, such as tests of normality and adjustment for multiple comparisons
☐	☒	A full description of the statistical parameters including central tendency (e.g. means) or other basic estimates (e.g. regression coefficient) AND variation (e.g. standard deviation) or associated estimates of uncertainty (e.g. confidence intervals)
☐	☒	For null hypothesis testing, the test statistic (e.g. *F*, *t*, *r*) with confidence intervals, effect sizes, degrees of freedom and *P* value noted<br>*Give P values as exact values whenever suitable.*
☒	☐	For Bayesian analysis, information on the choice of priors and Markov chain Monte Carlo settings
☒	☐	For hierarchical and complex designs, identification of the appropriate level for tests and full reporting of outcomes
☒	☐	Estimates of effect sizes (e.g. Cohen's *d*, Pearson's *r*), indicating how they were calculated

Our web collection on statistics for biologists contains articles on many of the points above.

Software and code

Policy information about availability of computer code

Data collection	IF images:<br>- Leica LASX software v 3.5.7.23225.<br><br>scRNA-seq:<br>- libraries were sequenced and fastQ files were obtained from DNBSEQ-T7 sequencer and software.<br><br>TEM:<br>- MyTEM, MyMesh, MyStitch (Faas et al. - Virtual nanoscopy: Generation of ultra-large high resolution electron microscopy maps - JCB 2012)<br><br>SBF-SEM:<br>- SBF-SEM image stacks were acquired as dm4 files and aligned using Digital Micrograph software (Gatan).
Data analysis	IF image analysis:<br>- Leica LASX software v 3.5.7.23225.<br>- Imaris Software x64 9.5.0<br><br>scRNAseq:<br>- Demultiplexing according to the sample barcodes, and subsequent read alignment were performed using Cell Ranger (10x Genomics, v3.1.0)<br>- Raw unfiltered data matrices from the Cell Ranger output were further processed with R (v4.1.1) and Seurat package (v4.0.4)<br>- Heatmaps displaying gene expression of ligand and receptors were prepared with the ComplexHeatmap package (v2.11.1) in R<br>- All other heatmaps were prepared with BIOMEX software (v1.5 - https://carmelietlab.sites.vib.be/en/biomex)<br>- DGEA outputs were used for GSEA using the ClusterProfiler package (v4.0.5)<br>- For L/R analysis, we used CellChat package (v1.1.3)

TEM image analysis:
- Aperio ImageScope v12.4.3.5008

SBF-SEM:
- SBF-SEM datasets were annotated using artificial intelligence-based image segmentation by Ariadne (Ariadne-Service GmbH).
- Convolutional neural networks (CNNs) were implemented in the ELEKTRONN deep learning library (www.elektronn.org).
- Image stacks of segmented structures (~12 Gb or ~25 Gb in size) were binned (in X and Y) using Fiji v1.5 or IMOD 4.11 to decrease the file size, and converted to mrc stacks and the headers were corrected using IMOD.
- Cell types were annotated by hand according to morphology and location and surfaces were smoothed, simplified and visualized using AMIRA 3D v 2021.3 software for life sciences (Thermo-Fisher Scientific)
- Surfaces were imported into Cinema4D R.22 (Maxon) for final rendering using Redshift V.3 (Maxon).

For manuscripts utilizing custom algorithms or software that are central to the research but not yet described in published literature, software must be made available to editors and reviewers. We strongly encourage code deposition in a community repository (e.g. GitHub). See the Nature Portfolio guidelines for submitting code & software for further information.

Data

Policy information about availability of data

All manuscripts must include a data availability statement. This statement should provide the following information, where applicable:

- Accession codes, unique identifiers, or web links for publicly available datasets
- A description of any restrictions on data availability
- For clinical datasets or third party data, please ensure that the statement adheres to our policy

Both raw and processed sequencing data are available in ArrayExpress under accession number E-MTAB-11429 with username: Reviewer_E-MTAB-11429 and password: TVhjdkpi for reviewing purposes and will be made publicly available after publication.

Field-specific reporting

Please select the one below that is the best fit for your research. If you are not sure, read the appropriate sections before making your selection.

☒ Life sciences ☐ Behavioural & social sciences ☐ Ecological, evolutionary & environmental sciences

For a reference copy of the document with all sections, see nature.com/documents/nr-reporting-summary-flat.pdf

Life sciences study design

All studies must disclose on these points even when the disclosure is negative.

Sample size

No sample size calculation was performed.
For analysis of vascularization using IF, we aimed to collect >3 untransplanted and transplanted organoids per experiment.
For scRNAseq analysis, the targeted cell capture was 9000 cells per condition to enable evaluation of cell populations while ensuring adequate sequencing depth. The number of organoids used for dissociation was much higher than necessary for this number of cells, to mitigate for inter-organoid variability and because we used the remainder of the cells for a separate experiment.
For TEM, we analyzed 3 transplanted organoids from hiPSC-MAFB and 2 transplanted organoids from LUMC0072, to enable evaluation of variability between organoids and cell lines.
For SBF-SEM followed by 3D reconstruction, we analyzed 2 additional transplanted organoids from hiPSC-MAFB. This relatively new and labour-intensive technique did not allow for studying larger numbers of organoids.

Data exclusions

Data from 1 experiment in which organoids were transplanted on d7+14 instead of d7+11/12 for logistical reasons were excluded from the analysis, since a thorough comparison between organoids transplanted at this timepoint versus our standard timepoints was not performed.

Replication

Transplantation experiments were performed >10 times over the course of 4 years. In all experiments, organoids became vascularized upon transplantation, as measured through IF stainings and lectin injection.
scRNAseq data were derived from 1 experiment using the hiPSC-MAFB cell line. A total of 309 organoids were dissociated for this purpose to mitigate potential inter-organoid variability.
TEM data were derived from 2 separate experiments using a different cell line in each experiment (hiPSC-MAFB and LUMC0072) to ensure replicability in different cell lines.
SBF-SEM data were derived from 1 experiment using the hiPSC-MAFB cell line. 4 glomerular structures in 1 untransplanted organoid and 3 glomerular structures in 2 transplanted organoids were analyzed.

Randomization

For each experiment, organoids were randomly assigned for transplantation or in vitro culture

Blinding

Investigators were not blinded to allocation during analysis or experiments. Untransplanted and transplanted kidney organoids are easy to distinguish during IF and EM imaging as well as scRNA sequencing due to the presence of chicken derived cells and blood in transplanted organoids.

Reporting for specific materials, systems and methods

We require information from authors about some types of materials, experimental systems and methods used in many studies. Here, indicate whether each material, system or method listed is relevant to your study. If you are not sure if a list item applies to your research, read the appropriate section before selecting a response.

Materials & experimental systems

n/a	Involved in the study
☐	☒ Antibodies
☐	☒ Eukaryotic cell lines
☒	☐ Palaeontology and archaeology
☐	☒ Animals and other organisms
☒	☐ Human research participants
☒	☐ Clinical data
☒	☐ Dual use research of concern

Methods

n/a	Involved in the study
☒	☐ ChIP-seq
☒	☐ Flow cytometry
☒	☐ MRI-based neuroimaging

Antibodies

Antibodies used	Primary Antibodies and lectins<br>Nephrin - Sheep anti-Human (NPHS1) Polyclonal R&D Systems AF4269 1:100<br>CD31 - Mouse anti-Human Monoclonal BD biosciences 555444 1:100<br>E-cadherin - Mouse anti-Human (ECAD) Monoclonal BD biosciences 610181 1:300<br>Lotus Tetragonolobus Lectin (LTL) - Vector Laboratories B-1325 1:300<br>Lens Culinaris Agglutinin rhodamine (LCA) - Vector Laboratories RL-1042-5 1:1<br>Platelet-Derived Growth Factor Receptor β Mouse anti-Human (PDGFRβ) Monoclonal R&D Systems MAB1263 1:50<br><br>Secondary Antibodies<br>Alexa Fluor 568 - Donkey anti-Sheep IgG Polyclonal Thermo Fisher Scientific A-21099 1:500<br>Alexa Fluor 647 - Donkey anti-Sheep IgG Polyclonal Thermo Fisher Scientific A-21448 1:500<br>Alexa Fluor 405 - Donkey anti-Mouse IgG Polyclonal Abcam ab175658 1:500<br>Alexa Fluor 488 - Donkey anti-Mouse IgG Polyclonal Thermo Fisher Scientific A-21202 1:500<br>Streptavidin Alexa Fluor 532 conjugate - Thermo Fisher Scientific S11224 1:200<br>Streptavidin Alexa Fluor 647 conjugate - Thermo Fisher Scientific S21374 1:200
Validation	All antibodies and isotype controls were validated in human kidney samples.

Eukaryotic cell lines

Policy information about cell lines

Cell line source(s)	LUMC0072iCTRL01 (LUMC0072): reprogrammed from fibroblasts using non-integraring RNA (RNA Simplicon reprogramming kit Millipore), hiPSC core-facility, LUMC. Detailed information can be found at https://hpscreg.eu/<br>LUMC0020iCTRL6.4 (LUMC0020): reprogrammed from skin fibroblasts using non-integrating Sendai virus, hiPSC core-facility, LUMC. Detailed information can be found at https://hpscreg.eu/<br>hiPSC reporter MAFB:mTagBFP2 (hiPSC-MAFB): Targeted iPSC clones were derived from male fibroblast line (ATCC # CRL2429) using a simultaneous reprogramming/gene targeting strategy. Episomal reprogramming vectors and gene targeting factors were introduced using Neon Transfection Device (1400V, 20ms, 2 pulses). Detailed information can be found in Howden et al. - Simultaneous reprogramming and gene editing of human fibroblasts - Nature Protocols 2018 and Howden et al - Simultaneous reprogramming and gene correction of patient fibroblasts - Stem Cell Reports 2015
Authentication	We did not authenticate cell lines at DNA level. New vials from the working cell bank were regularly thawed. For the MAFB cell line, we were able to confirm the identity of the cell line through expression of BFP by podocytes.
Mycoplasma contamination	All cell lines tested negative for Mycoplasma contamination.
Commonly misidentified lines<br>(See ICLAC register)	none

Animals and other organisms

Policy information about studies involving animals; ARRIVE guidelines recommended for reporting animal research

Laboratory animals	Fertilized White Leghorn eggs (Gallus gallus domesticus, Drost Loosdrecht B.V.), Chicken embryos incubation day 0-12.
Wild animals	Study did not involve wild animals.
Field-collected samples	Study did not involve samples collected from the field.
Ethics oversight	Under Dutch law, not ethical approval is required for experiments using chicken embryos

Note that full information on the approval of the study protocol must also be provided in the manuscript.
